# Metabolomic landscape of macrophage discloses an anabolic signature of dengue virus infection and antibody-dependent enhancement of viral infection

**DOI:** 10.1371/journal.pntd.0011923

**Published:** 2024-02-02

**Authors:** Li Xu, Min Li, Jingpu Zhang, Dongxiao Li, Jie Tao, Fuchun Zhang, Xia Jin, Jiahai Lu, Tiefu Liu

**Affiliations:** 1 Scientific Research Center, Shanghai Public Health Clinical Center, Fudan University, Shanghai, China; 2 Metabo-Profile Biotechnology Company, Shanghai, China; 3 Guangzhou Eighth People’s Hospital, Guangzhou Medical University, Guangzhou, Guangdong, China; 4 Key Laboratory for Tropical Disease Control, School of Public Health, Sun Yat-sen University, Guangzhou, Guangdong, China; 5 One Health Center of Excellence for Research & Training, Sun Yat-Sen University, Guangzhou 510080, China; 6 National Medical Products Administration Key Laboratory for Quality Monitoring and Evaluation of Vaccines and Biological Products, Guangzhou 510080, China; 7 Hainan Key Novel Thinktank "Hainan Medical University ’One Health’ Research Center", Haikou 571199, China; 8 Institute of One Health, Wenzhou Medical University, Wenzhou, Zhejiang, 325035, China; KU Leuven, BELGIUM

## Abstract

Dengue virus (DENV) infection causes dengue fever, the most prevalent arthropod-transmitted viral disease worldwide. Viruses are acellular parasites and obligately rely on host cell machinery for reproduction. Previous studies have indicated metabolomic changes in endothelial cell models and sera of animal models and patients with dengue fever. To probe the immunometabolic mechanism of DENV infection, here, we report the metabolomic landscape of a human macrophage cell model of DENV infection and its antibody-dependent enhancement. DENV infection of THP-1-derived macrophages caused 202 metabolic variants, of which amino acids occupied 23.7%, fatty acids 21.78%, carbohydrates 10.4%, organic acids 13.37%, and carnitines 10.4%. These metabolomic changes indicated an overall anabolic signature, which was characterized by the global exhaustion of amino acids, increases of cellular fatty acids, carbohydrates and pentoses, but decreases of acylcarnitine. Significant activation of metabolic pathways of glycolysis, pentose phosphate, amino acid metabolism, and tricarboxylic acid cycle collectively support the overall anabolism to meet metabolic demands of DENV replication and immune activation by viral infection. Totally 88 of 202 metabolic variants were significantly changed by DENV infection, 36 of which met the statistical standard (P<0.05, VIP>1.5) of differentially expressed metabolites, which were the predominantly decreased variants of acylcarnitine and the increased variants of fatty acids and carbohydrates. Remarkably, 11 differentially expressed metabolites were significantly distinct between DENV only infection and antibody-dependent enhancement of viral infection. Our data suggested that the anabolic activation by DENV infection integrates the viral replication and anti-viral immune activation.

## Author summary

Dengue fever, a widespread viral disease transmitted by mosquitoes, is caused by the dengue virus (DENV). In our study, we explored how DENV infection affects human macrophages, crucial immune cells in our body. By analyzing the metabolic changes within these cells during DENV infection and its antibody-dependent enhancement, we uncovered a metabolic landscape characterized by significant alterations.

Our findings revealed a shift towards utilization of amino acids and increased production of carbohydrates and fatty acids, indicating an overall boost in cellular activity—a signature response to the virus’s replication demands and immune system activation triggered by the infection. Specifically, we identified 88 metabolic changes induced by DENV, with 36 showing notable statistical significance, highlighting decreased acylcarnitine and increased fatty acids and carbohydrates as prominent alterations. Importantly, our study identified 11 distinct metabolites significantly different between DENV infection alone and its enhancement by antibodies. These results strongly suggest that the virus not only facilitates its replication but also orchestrates anabolic processes that integrate viral growth with the host immune responses.

Understanding these metabolic dynamics during DENV infection could offer valuable insights for potential therapeutic interventions in combating this prevalent and impactful disease.

## Introduction

Dengue fever is the most prevalent arthropod-transmitted viral disease worldwide and the incidence has risen sharply in recent decades [1]. Over the past 20 years, the number of dengue cases reported to WHO has increased more than eightfold, approximately 3.9 billion people in 129 countries are at risk of dengue virus (DENV) infection with 70% of the infected population in Asia [2,3].

DENV is the causative agent of dengue fever and contains 4 different serotypes (DENV-1, DENV-2 DENV-3, and DENV-4). They naturally infect mononuclear phagocyte lineage including monocytes, macrophages, and dendritic cells (DCs) [4,5] via the cell-surface molecules or receptors. Their infection causes disease symptoms ranging from dengue fever (DF), a form of self-limited febrile illness, to dengue hemorrhagic fever (DHF) and dengue shock syndrome (DSS) [6]. Viral virulence is an important risk factor for severe disease, and certain DENV-2 and DENV-3 genotypes are more often associated with DHF [7–10], in particular, the Southeast Asia genotype of DENV-2 replicates to higher titers in human macrophages and DCs [11,12] and may lead to increased viral pathogenicity in humans [9].

Primary infection of a single serotype of DENV tends to cause mild dengue fever, while the secondary infection with a heterotypic DENV serotype was found to be associated with severe dengue disease. The mechanism of severe dengue disease is not fully understood and is currently hypothesized by extrinsic and intrinsic antibody-dependent enhancement (ADE) theories. The extrinsic ADE theory states that antibodies produced during primary DENV infection cross-bind to secondary infected heterologous dengue viruses to form a viral antigen-antibody complex that facilitates virus entry by binding to IgG Fcγ receptor (FcγR) on the cell surface, resulting in high viral load infection that aggravates disease severity. The intrinsic ADE theory suggests that FcγR-mediated viral infection modulates intracellular immune effectors that promote virus replication and release [13,14]. Furthermore, antibody-dependent DENV infection activates intracellular signaling pathways distinct from canonical receptor-mediated endocytosis, increasing the expression of host dependency factors associated with RNA splicing, mitochondrial respiratory chain function, intracellular vesicle transport rate, and ribosome gene expression [15,16].

Viral infection induces an integrative process of innate and adaptive immune responses that couples metabolism in terms of energy support and building blocks supply [17]. Metabolites yielded in cellular metabolism are the biochemical footprints capable of revealing the state of the cell in a specific time frame, and the metabolomics analysis is one of the approaches to disclose metabolite alternations and dissect intracellular molecular mechanisms of pathophysiological activities. Since the first metabolomic study on serum samples from patients with primary DENV infection in 2013 [18], several metabolomics studies have been performed on various samples of cell model [19] and animal model [20] of DENV infection and samples of patients with DF, DHF and/or DSS [21–24] to explore metabolic mechanism, diagnostic biomarkers, and druggable metabolic targets of DENV infections. Although the metabolomic findings vary in different studies due to the differences in cell type, disease stage, genetic and nutrition background, the majorly altered metabolites were identified as free fatty acids, acylcarnitine, phospholipids, amino acids, purines, pyrimidine, bile acids from different metabolic pathways, particularly the lipid metabolic pathways.

Despite several previous metabolomics studies on DENV infection, little is known about the alterations occurring in intracellular metabolic pathways in human immune cells during DENV infection. Particularly, human monocytes and macrophages, which are the main targets of natural DENV infections, play a central role during DENV immunopathogenesis, as well as host anti-viral immunity [25–28]. Elucidating the comprehensive metabolic changes in monocytes/macrophages may shed light on the immunometabolic mechanisms involved in immune cell responsiveness upon DENV infection.

To probe the immunometabolic mechanism of DENV infection, we thus studied the metabolomic profiles of DENV infection in immune cells using a human monocytic cell model of DENV infection under non-ADE and ADE conditions. The general outcomes disclosed an anabolic signature of dengue virus infection, which is evidenced by the increases in lipid biosynthesis, carbohydrates, and pentoses and the exhaustion of amino acids and acylcarnitine.

## Materials and methods

### Cells and virus

THP-1 cells were grown in Roswell Park Memorial Institute 1640 (RPMI-1640, Gibco, NY, USA) medium supplemented with 10% heat-inactivated fetal bovine serum (FBS, Gibco, Auckland, New Zealand), 50 U/ml penicillin and 50 μg/ml streptomycin (Gibco, NY, USA) at 37°C with 5% CO_2_. THP-1 monocytes were differentiated into macrophages by incubation with 100 ng/ml phorbol 12-myristate 13-acetate (PMA) for 48 h. DENV-2 (strain 16681, provided by Dr. Claire Huang, Centers for Disease Control and Prevention, Ft. Collins, CO, USA) was grown in C6/36 *Aedes albopictus* cells and titrated on Vero cells by a focus-forming assay.

### DENV infection in macrophages

For DENV only infection (non-ADE), DENV-2 was added to THP-1-derived macrophages (MOI = 20) for 2 h. Infected cells were then washed once and cultured in RPMI 1640 medium supplemented with 10% FBS for 24 h. Cells were detached from plates by EDTA treatment and washed with PBS for further metabolomic analysis (six replicates). For ADE infection, the heat-inactivated convalescent serum from a patient with DENV-3 infection was diluted at 1:4000 and then incubated with the same volume of DENV-2 (6×10^7^ PFU/ml) for 1 h at room temperature. The virus/serum mixture was subsequently added to THP-1-derived macrophages (MOI = 20) for 2 h at 37°C. Infected cells were then washed once and cultured in RPMI 1640 medium supplemented with 10% FBS for 24 h at 37°C. Cells were collected for further analysis of metabolome profiles (six replicates). To detect DENV infectivity, infected cells were collected and fixed with 4% PFA. Viral replication was evaluated by intracellular staining with DENV-2 NS1 immunized mouse serum [29] for 30 min at 4°C, followed by incubation with goat anti-mouse IgG conjugated to Alexa Fluor 568 (Abcam, Cambridge, UN) for another 30 min at 4°C. The percentage of infected cells was determined by flow cytometry using an LSRFortessa (BD Biosciences, USA) and analyzed using FlowJo software version 10.4.

### Targeted metabolomics quantification

The targeted metabolomics quantification analysis was performed using Q300 Kit (Metabo-Profile, Shanghai, China) according to a detailed procedure [30]. The Q300 kit detects multiple trans-quantitative metabolites such as amino acids, phenols, phenyl or benzyl derivatives, indoles, organic acids, fatty acids, sugars and bile acids in biological samples on the same microtiter plate. This kit adopts L_Arginine_15N2, Hippuric acid_D5, TCDCA_D9, D_Glucose_D7, Carnitine_D3, C5 0_D9, Citric acid_D4, one-to-one standard, and other internal standards. The method has been patented (CN109298115B), published [30], and cited by other studies [31–33]. To validate the stability and reproducibility of the chromatographic separation of low concentrations of metabolites that may have large coefficient of variation (CV) values, we performed Principal component analysis (PCA) on quality control (QC) samples at the same time, the first principal component (PC1) results indicated overall stable of the QC (**S1 Fig**).

#### Chemicals and preparation of stock solutions

Metabolite compound standards were obtained from Sigma-Aldrich (St. Louis, MO, USA), Steraloids Inc. (Newport, RI, USA) and TRC Chemicals (Toronto, ON, Canada). The stock solution for each compound was prepared by dissolving the individual standard in water, methanol, sodium hydroxide, or hydrochloric acid at a concentration of 5.0 mg/mL, and calibration stock solution was made by mixing appropriate amount of each stock solution. Optima LC/MS grade formic acid was obtained from Sigma-Aldrich (St. Louis, MO, USA), and Optima LC/MS grade methanol, acetonitrile, and isopropanol were purchased from Thermo-Fisher Scientific (FairLawn, NJ, USA). Ultrapure water was produced by a Mill-Q Reference system equipped with a LC-MS Pak filter (Millipore, Billerica, MA, USA).

#### Metabolite extraction

About 5×10^6^ cells of each sample in safelock Eppendorf tube were mixed with pre-chilled zirconium oxide beads in 20 μL of deionized water and were homogenized for 3 min. Metabolites were extracted by adding 150 μL of methanol containing internal standard and homogenizing for another 3 min, the homogenate was centrifugated at 18000 g for 20 min. The supernatant was transferred to a 96-well plate and subjected to following procedures on an Eppendorf epMotion Workstation (Eppendorf Inc., Humburg, Germany): 20 μL of freshly prepared derivative reagents were added to each well, then, 96-well plate was sealed, derivatized at 30°C for 60 min, evaporated for 2 h, and reconstituted by adding 330 μL of ice-cold 50% methanol. The plate was then cold-down at -20°C for 20 min and followed by 4000 g centrifugation at 4°C for 30 min. 135 μL of supernatant was transferred to a new 96-well plate with 10 μL internal standards in each well. Serial dilutions of derivatized stock standards were added to blank wells, and the plate was sealed for LC-MS analysis within 48 h.

#### UPLC-MS/MS quantitative analysis of metabolites

The contents of the intermediate metabolites were quantitatively determined using an ultra-performance liquid chromatography coupled to tandem mass spectrometry (UPLC-MS/MS) system (ACQUITY UPLC-Xevo TQ-S, Waters Corp., Milford, MA, USA). A comprehensive set of rigorous quality control/assurance (QC/QA) procedures was employed to ensure a consistently high quality of analytical results, including quality controls of the test mixtures, internal standards, pooled biological samples, conditioning samples, and solvent blank samples. The cell samples were analyzed in random group pairs, together with QC samples, calibrators, and blank samples, to diminish analytical bias. Metabolite concentration in samples was determined by calibration curve of standard samples.

For quality control of metabolomic analysis, the test mixtures of standards with a mass profile covering the mass range used for sample analysis were analyzed at the beginning and end of each batch run to ensure instrument performance within laboratory specifications (retention time stability, chromatographic peak shape, and peak signal intensity). The retention time shift was within 4 sec and the difference of peak intensity was within 15% for LC-MS. The internal standards were added to the test samples in order to monitor analytical variations during the entire sample preparation and analysis processes. The pooled QC samples were prepared by mixing aliquots of the study samples and were injected at regular intervals (after every 14 test samples for LC-MS) throughout the analytical run. Reagent blank samples were the mixture of solvents used for sample preparation and were also used to wash the column and remove cumulative matrix effects throughout the study. The calibrators consisted of a blank sample (matrix sample without internal standard), a zero sample (matrix sample with internal standard), and a series of seven concentrations covering the expected range for the metabolites in cell samples. The lower limit of quantification (LLOQ) and the upper limit of quantification (ULOQ) were the lowest and highest concentrations of the standard curve that were measured with acceptable accuracy and precision using Q300 Kit [30] as described above. In brief, the accuracy of quantitation was assessed by determining the recoveries of standard metabolites, and recovery ranging from 80% to 120% and CV less than 15% was defined as acceptable accuracy. All metabolite concentration was normalized as pmol/million cells.

### Statistics

The raw data generated by UPLC-MS/MS were processed for peak integration, calibration, and quantitation using MassLynx software (v4.1, Waters, Milford, MA, USA). The multivariate statistical analyses including principal component analysis (PCA), partial least square discriminant analysis (PLS-DA), orthogonal partial least square discriminant analysis (OPLS-DA), random forest, and support vector machine learning and the univariate statistical analyses including student t-test, Mann-Whitney-Wilcoxon (U-test), ANOVA, and correlation analysis were performed by iMAP platform (v1.0; MetaboProfile, Shanghai, China). The variable importance in projection (VIP) was obtained based on the OPLS-DA model, and metabolites with VIP ≥ 1 and P value <0.05 (univariate analyses were based on whether the data were normally distributed) were determined as statistically significant. The Z-Score, also known as the standard score, was used to determine how many standard deviations an entity is away from the mean of the control group. Arbitrary fold change (FC) cut-offs of >2 and significance p-values of <0.05 were used to judge the significant difference of differentially expressed metabolites. To investigate the biological meaning of differentially expressed metabolites, metabolite-associated pathway enrichment analysis was performed using MetaboAnalyst 4.0 with KEGG(Kyoto Encyclopedia of Genes and Genomes)database [34]. A threshold of p < 0.05 was used for considering the function or pathway to be impactful. P values were determined by two different pathway analysis methods: the hypergeometric test [35] and the pathway impact value [36].

## Results

### Establishment of a cell model for DENV and ADE infections

To elucidate the intracellular metabolic characteristics of human immune cells after DENV infections, we performed the DENV infection assay in the absence or presence of DENV-immune sera, using a monocyte/macrophage-like cell line THP-1, which has been widely used to study the intracellular mechanisms of DENV infection [37–39]. This cell line is susceptible to DENV infection, and also expresses FcγRI and FcγRII on the cell surface [40], representing a suitable cell-based model for DENV and ADE infections.

**Fig 1:**
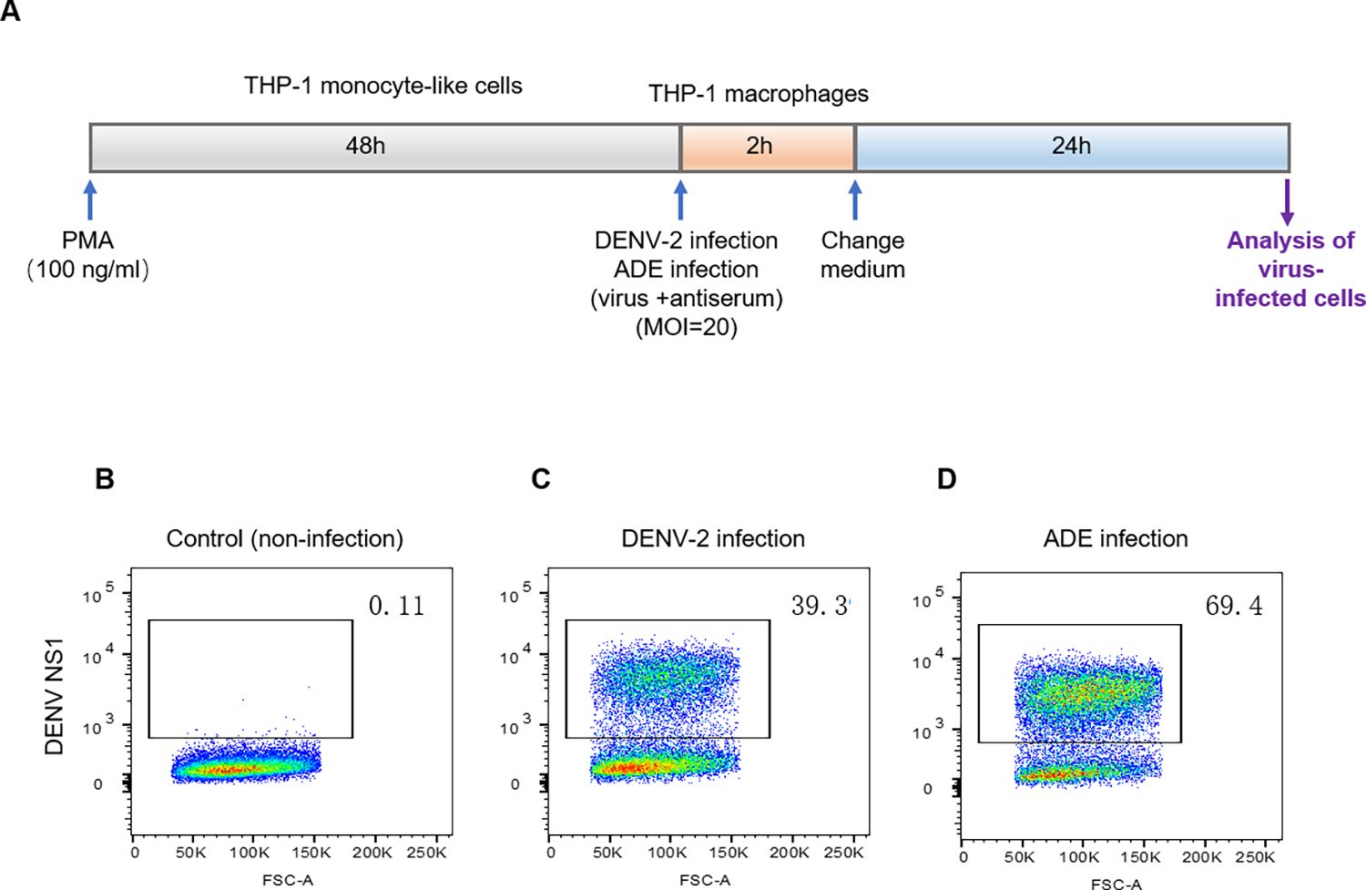
THP-1 derived macrophage cell model for DENV and ADE infections. THP-1 derived macrophages were infected with DENV-2 only or DENV-2 plus anti-DENV-3 serum, and intracellular viral antigens were stained with anti-DENV NS1 antibody and were evaluated by flow cytometry analysis. **A**: depiction of viral infection. **B**: non-viral infection. **C**: DENV-2 infection only. D: DENV-2 infection in the presence of anti-DENV3 serum. Representative data of three independent experiments were shown.

The cell model of DENV only infection (non-ADE) and ADE infection was established as schemed in **Fig 1A.** Intracellular viral antigens were stained with anti-DENV NS1 antibody and were analyzed by flow cytometry. In comparison with the mock-infected cells (**Fig 1B**), the DENV-2 only infection resulted in a cell infection rate of 39.3% (**Fig 1C**), while in the presence of antiserum (ADE), infected cells were increased up to 69.4% (**Fig 1D**), which was about twice as high as virus only infection.

### Overview of metabolic changes of dengue virus infection

**Fig 2:**
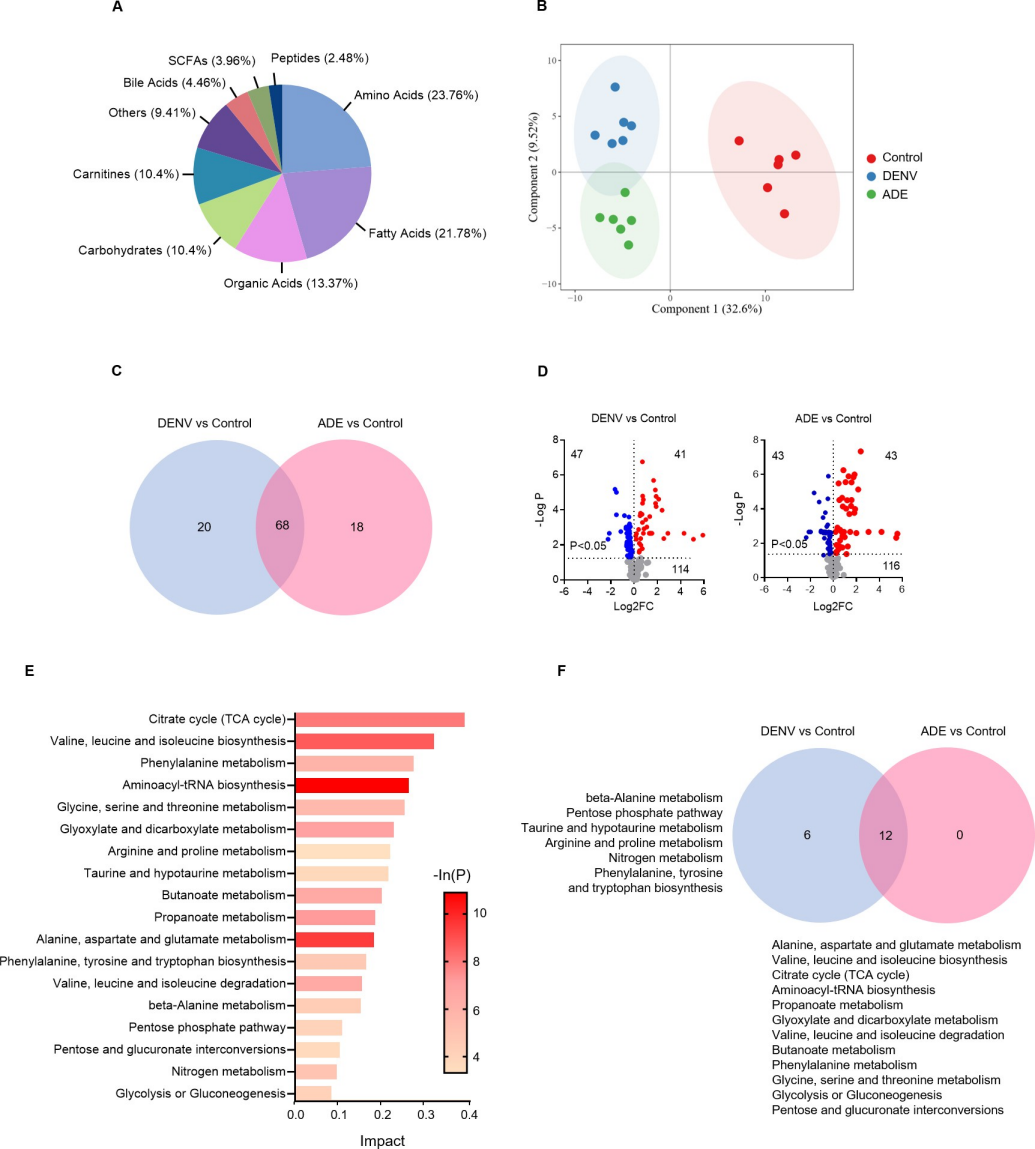
Overall metabolomic changes of THP-1-derived macrophages by DENV or ADE infections. The overall changes in metabolites and metabolic pathways by DENV or ADE infections were analyzed using targeted metabolomics quantification analysis. **A**: distributions of metabolic variants. **B**: unsupervised principal component analysis (PCA). **C**: differentially expressed metabolites of DENV-2 or ADE infection based on multidimensional statistical analysis. **D**: overall changes of metabolites by DENV-2 or ADE infection. **E**: heatmap of significantly changed metabolic pathways by DENV-2 or ADE infection. The mean Z-score of sextuplicate in each group was shown. **F**: overall changes in metabolic pathways by DENV-2 or ADE infection.

Intracellular metabolomic changes after virus-free (control), DENV-2 only infection (non-ADE), or ADE infection (DENV-2-antiserum complex) were quantitatively analyzed by ultra-high liquid chromatography-tandem mass spectrometry. A total of 202 altered metabolites were detected, of which were amino acids (23.76%), fatty acids (21.78%), organic acids (13.37%), carbohydrates (10.4%), and carnitines (10.4%) (**Fig 2A and S1 Table**). Metabolite class statistics showed that amino acid contents displayed the most metabolite detection frequency (62.17%), which was followed by carbohydrates (18.53%), organic acids (10.51%), nucleotides (6.86%), fatty acids (1.47%) and others (0.46%) (**S2 Fig**). Unsupervised principal component analysis (PCA) showed well sampling reproducibility, significant aggregation of individual samples across groups, and significant differences among groups (**Fig 2B**). Multidimensional statistical analysis displayed the presence of differentially expressed metabolites between groups (**Fig 2C and S2 Table**). In comparison with the non-infected group, DENV-2 only infection resulted in significant changes in 88 metabolites, of which 41 metabolites were significantly elevated and 47 were significantly reduced; ADE infection resulted in significant changes in 86 metabolites, including 43 elevated and 43 decreased (**Fig 2D**).

The variation of metabolites by DENV infection was significantly involved in the changes of activities of 18 metabolic pathways (P<0.05) (**Fig 2E**). Most of them are related to the metabolism of amino acids, glycolysis, pentose phosphate and oxidative response (the tricarboxylic acid cycle). 12 of the 18 varied pathways were shared by non-ADE and ADE infections, the rest 6 varied pathways were specific to non-ADE infection, which were beta-alanine metabolism, pentose phosphate pathway, taurine and hypotaurine metabolism, arginine and proline metabolism, nitrogen metabolism, phenylalanine, tyrosine, and tryptophan biosynthesis (**Fig 2F**). The differences in metabolic pathways may distinguish metabolic prediction of non-ADE from ADE infections.

Among the changed metabolic pathways, the anabolic pathways of aminoacyl-tRNA (P = 1.86E-05) and alanine, aspartate, and glutamate metabolism (P = 8.07E-05) were the most significant, highlighting the anabolism of viral proteins (**Fig 2E**). Other highly activated metabolic pathways were glycolysis that supports energy demand during infection process, the pentose phosphate pathway, a branch of the glycolysis that provides ribose for nucleic acid synthesis, and the tricarboxylic acid cycle reactions that converges aerobic oxidation of metabolic substrates (sugars, fatty acids and amino acids), as well as the mutual transformation between intermediate metabolites, which is crosslinked with metabolism pathways of amino acids, glucose, and fatty acids.

In general, all these metabolites change collectively accord with a highly anabolic response to DENV infection.

### Dengue virus infection significantly consumes cellular amino acids

**Fig 3:**
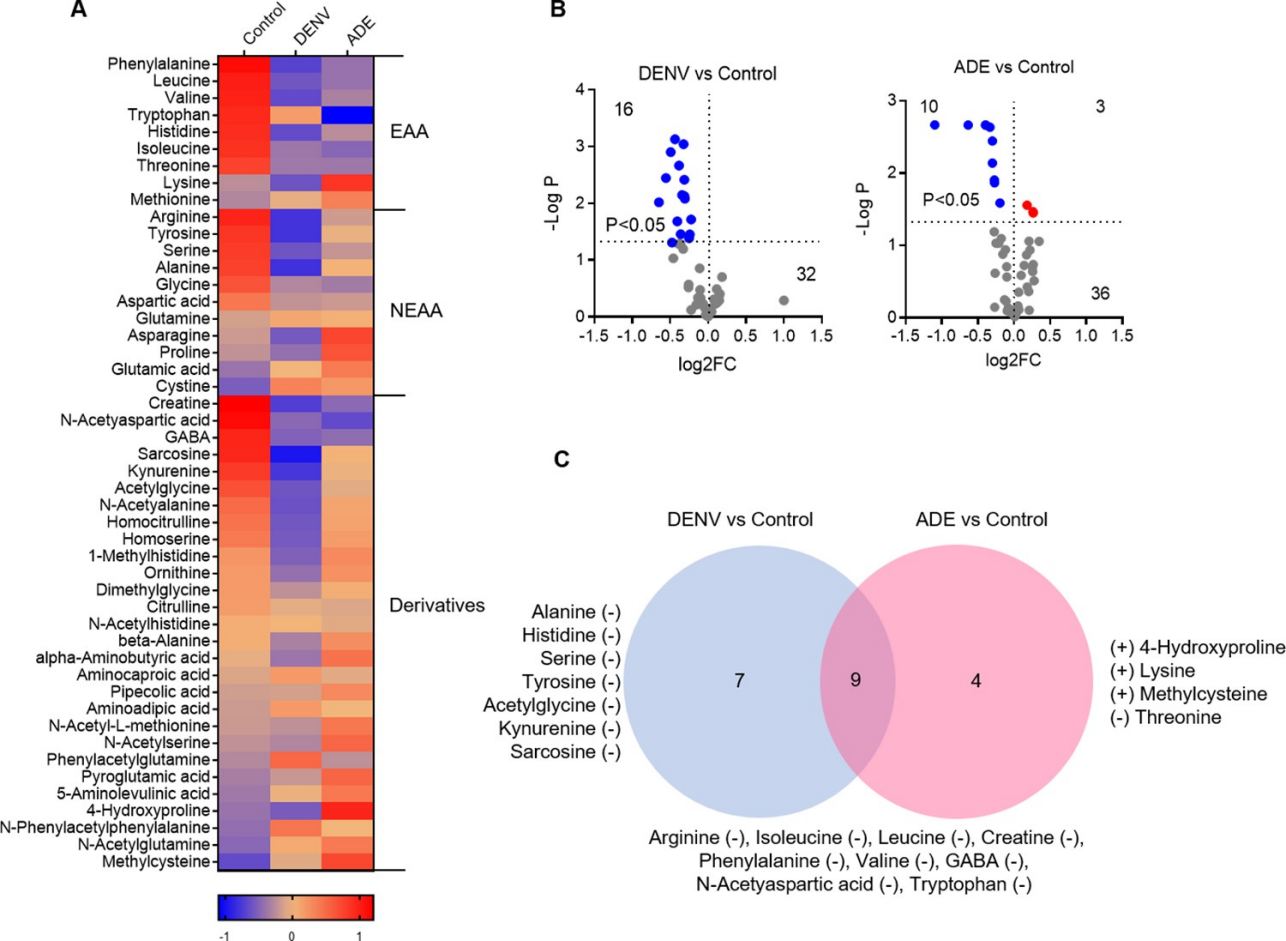
Dengue virus infection significantly consumes cellular amino acids. **A**: heatmap of amino acid variants. The mean Z-score of sextuplicate in each group was shown. **B**: amino acid variants by DENV infection only or ADE infection in comparison with non-viral infection. The mean density of sextuplicate of each variant was shown. Blue dot: significantly decreased variants; red dot: significantly increased variants. **C**: the distribution of significant amino acid variants under different infection conditions.

As indicated in **Fig 2E**, enrichment analysis of pathways reveals a relatively numerous enrichment of amino acid pathways, including essential amino acids such as valine, leucine, isoleucine, phenylalanine, tryptophan, and histidine. This suggests that amino acids have a substantial impact. DENV infection changed the cellular contents of 48 amino acids and their derivatives (**Fig 3A**), 20 of which reached significant (fold change (FC) >2, P<0.05) including 16 decreases in DENV only infection, 10 decreases and 3 increases in ADE infection (**Fig 3B**). These significant variants included 8 essential amino acids (histidine, isoleucine, leucine, lysine, phenylalanine, threonine, tryptophan, and valine), 3 conditionally non-essential amino acids (arginine, serine, and tyrosine), 1 non-essential amino acids (alanine), and 8 amino acid derivatives (4-hydroxyproline, acetylglycine, creatine, GABA, kynurenine, methylcysteine, N-acetyaspartic acid, and sarcosine). Among them, 7 variants were specifically produced by DENV only, 4 by ADE, and 9 were co-produced by both DENV only and ADE infections (**Fig 3C**). Most of their contents were significantly reduced, except that 3 (4-hydroxyproline, lysine, and methylcysteine) were elevated by ADE infection (**S3 Fig**).

### Dengue virus infection generally increases cellular carbohydrate contents

**Fig 4:**
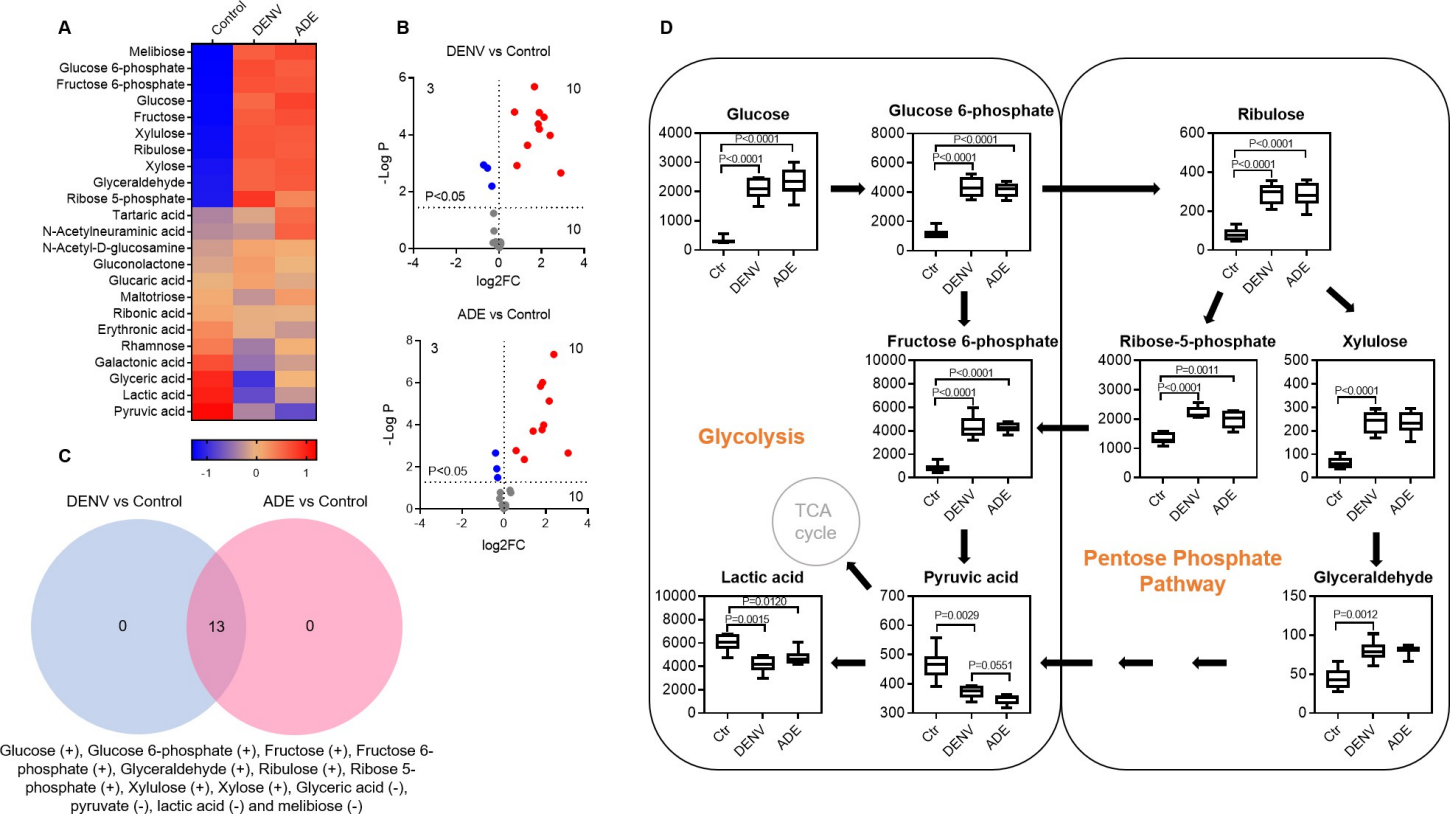
Dengue virus infection generally increases cellular carbohydrate contents. **A**: heatmap of carbohydrate variants. The mean Z-score of sextuplicate in each group was shown. **B**: display of carbohydrate variants by DENV infection only or ADE infection in comparison with non-viral infection. The mean density of sextuplicate of each variant was shown. Blue dot: significantly decreased variants; red dot: significantly increased variants. **C**: distribution of significant carbohydrate variants under different infection conditions. **D**: scheme of interplay of carbohydrate variants in glycolysis and pentose phosphate pathways.

Both non-ADE and ADE of DENV infections resulted in 23 carbohydrate variants (**Fig 4A**) and significantly co-produced 13 variants (FC>2, P<0.05), including one 12-carbon compound (melibiose), four 6-carbon compounds (glucose, glucose 6-phosphate, fructose, and fructose 6-phosphate), four 5-carbon compounds (ribulose, ribose 5-phosphate, xylulose, xylose), and four 3-carbon compounds (glyceraldehyde, glyceric acid, pyruvate, and lactic acid) (**Fig 4B and 4C**). The contents of glyceric acid, pyruvate, lactic acid and melibiose were significantly decreased, whereas, the others were significantly increased. These variants are the primary components of glycolysis and pentose phosphate pathways (**Figs 4D and S4**).

### Dengue virus infection significantly increases cellular fatty acid contents

**Fig 5:**
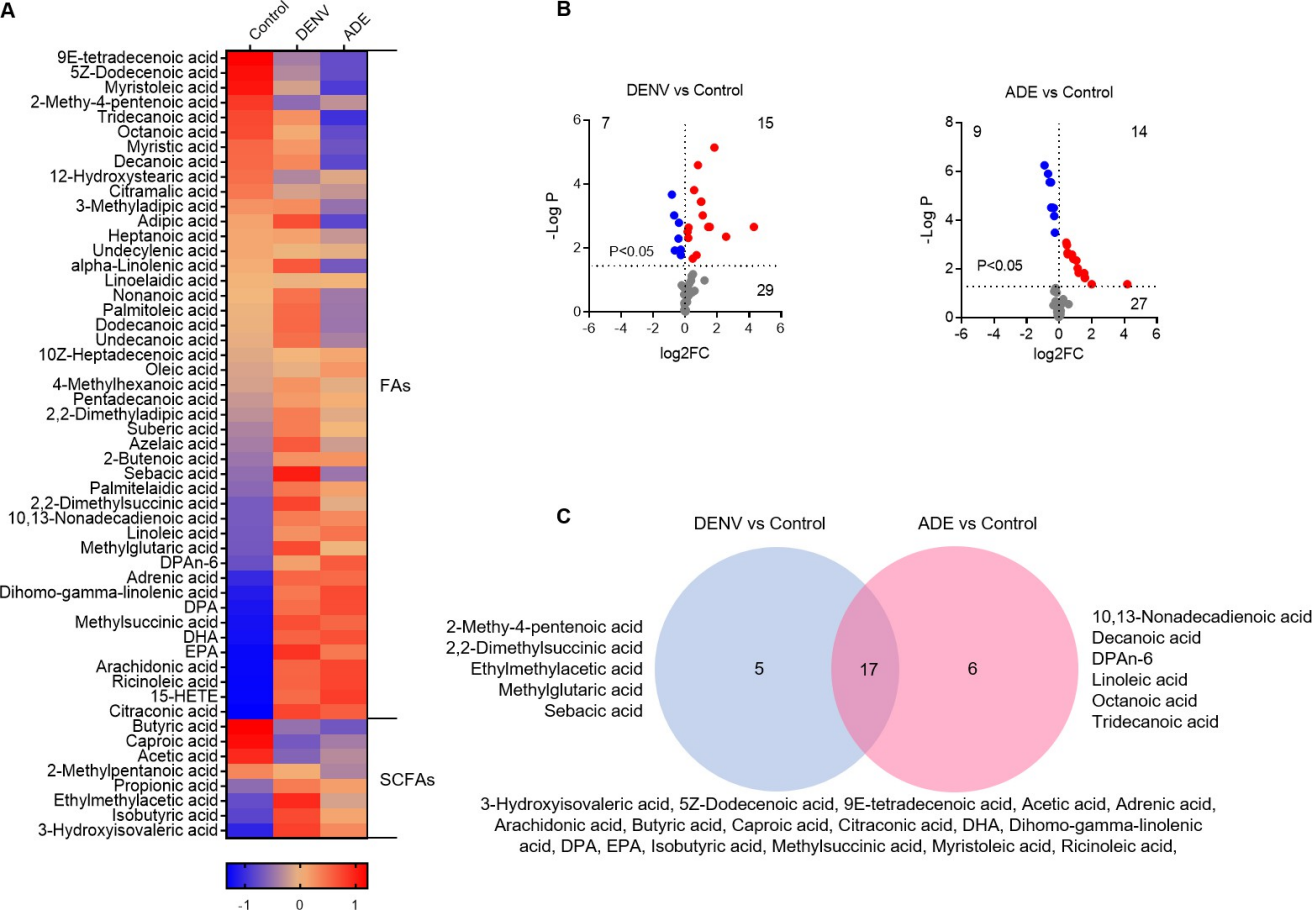
Dengue virus infection significantly increases cellular fatty acid contents. **A:** heatmap of fatty acid variants. The mean Z-score of sextuplicate in each group was shown. **B**: display of fatty acid variants by DENV infection only or ADE infection in comparison with non-viral infection. The mean density of sextuplicate of each variant was shown. Blue dot: significantly decreased variants; red dot: significantly increased variants. **C**: distribution of significant fatty acid variants under different infection conditions.

Dengue virus infection resulted in 44 variants of long-chain fatty acids and 9 variants of short-chain fatty acids (SCFA), of them 28 variants reached significant (FC>2, P<0.05) (**Fig 5A**). 7 variants were decreased and 15 variants were increased by DENV only infection, whereas, 9 were decreased and 14 were increased by ADE infection (**Fig 5B**). DENV only infection specifically produced 5 variants (2-methy-4-pentenoic acid, 2,2-Dimethylsuccinic acid, methylglutaric acid, ethylmethylacetic acid, and sebacic acid), while ADE infection specifically produced 6 variants (decanoic acid, octanoic acid, linoleic acid, DPAn-6, 10,13-nonadecadienoic acid, and tridecanoic acid). The rest 17 variants were produced by both non-ADE and ADE infections (**Fig 5C**). Remarkably, 6 shared variants (adrenic acid, arachidonate, dihomo-gamma-linolenate, DHA, DPA, and EPA) participate in the pathway of unsaturated fatty acids biosynthesis, and all were increased. Among the DENV only infection-specific variants, except for 2-methy-4-pentenoic acid, the other 4 variants were significantly increased (FC>2, P<0.05). However, among the 6 ADE-specific variants, 3 were significantly decreased (decanoic acid, octanoic acid, tridecanoic acid) and other 3 were increased (**S5 Fig**).

### Dengue virus infection significantly reduces cellular acylcarnitine contents

**Fig 6:**
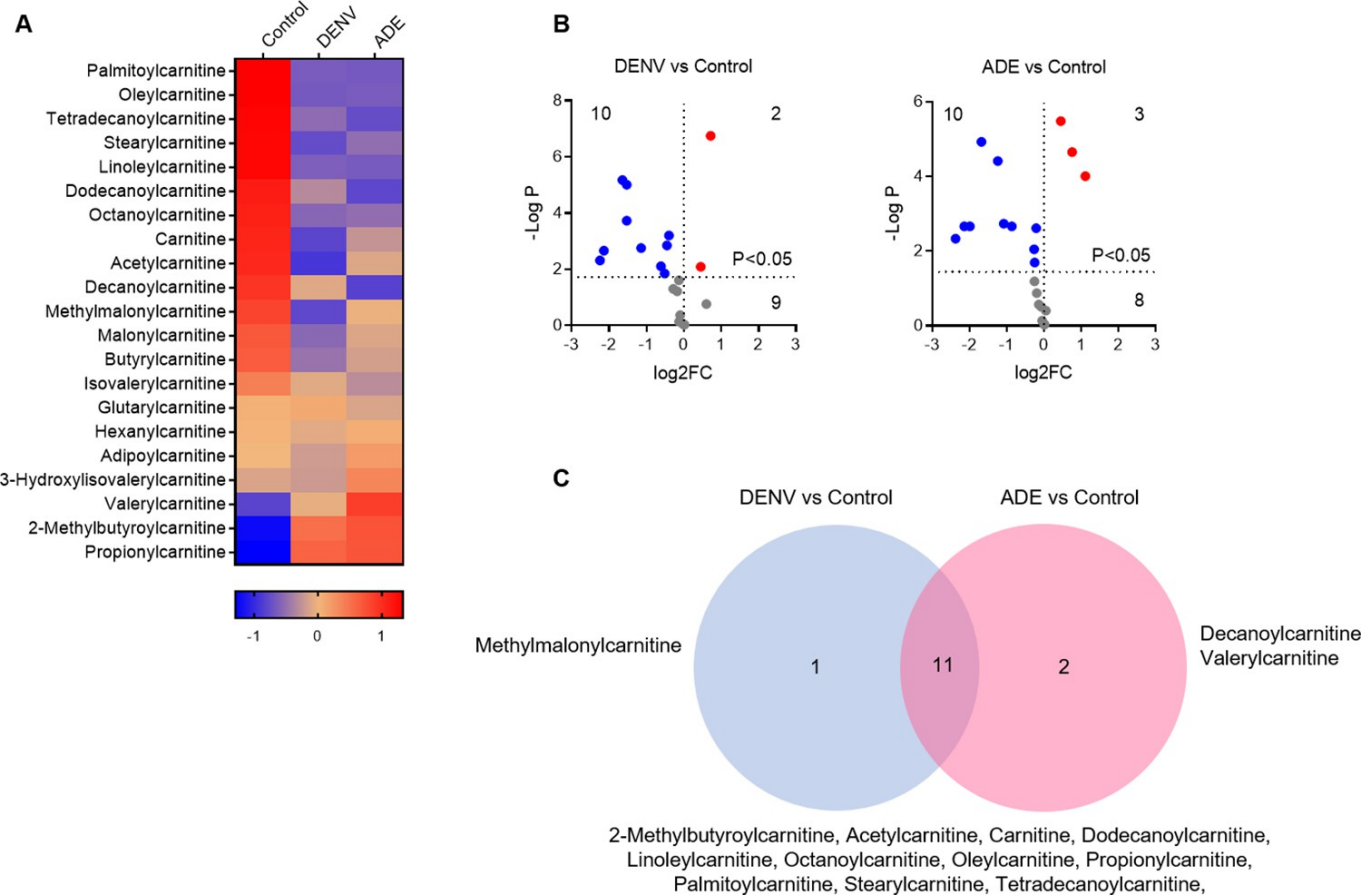
Dengue virus infection significantly reduces cellular acylcarnitine contents. **A:** heatmap of acylcarnitine variants. The mean Z-score of sextuplicate in each group was shown. **B**: display of acylcarnitine variants by DENV infection only or ADE infection in comparison with non-viral infection. The mean density of sextuplicate of each variant was shown. Blue dot: significantly decreased variants; red dot: significantly increased variants. **C**: distribution of significant acylcarnitine variants under different infection conditions.

Carnitine is a non-essential amino acid and a quaternary ammonium compound, and its fatty acid ester acylcarnitine plays a key role in lipid β-oxidation. DENV infection led to 21 carnitine variants, of them 14 carnitine variants were significant (FC >2, P<0.05) (**Fig 6A**). 10 variants were decreased and 2 increased by DENV only infection, whereas 10 variants were decreased and 3 increased by ADE infection (**Fig 6B**). One variant (methylmalonylcarnitine) was specific to DENV only infection, while two variants (dodecanoylcarnitine and valerylcarnitine) were specific to ADE infection (**Fig 6C**). The content of DENV only infection-produced methylmalonylcarnitine was significantly decreased, whereas, the content of ADE infection-produced dodecanoylcarnitine was reduced but valerylcarnitine was increased (**S6 Fig**). The general reduction of carnitine contents indicates inhibition of fatty acid catabolism that supports lipid anabolism to meet the need of active viral replications.

### Effect of dengue virus infection on cellular organic acid contents

**Fig 7:**
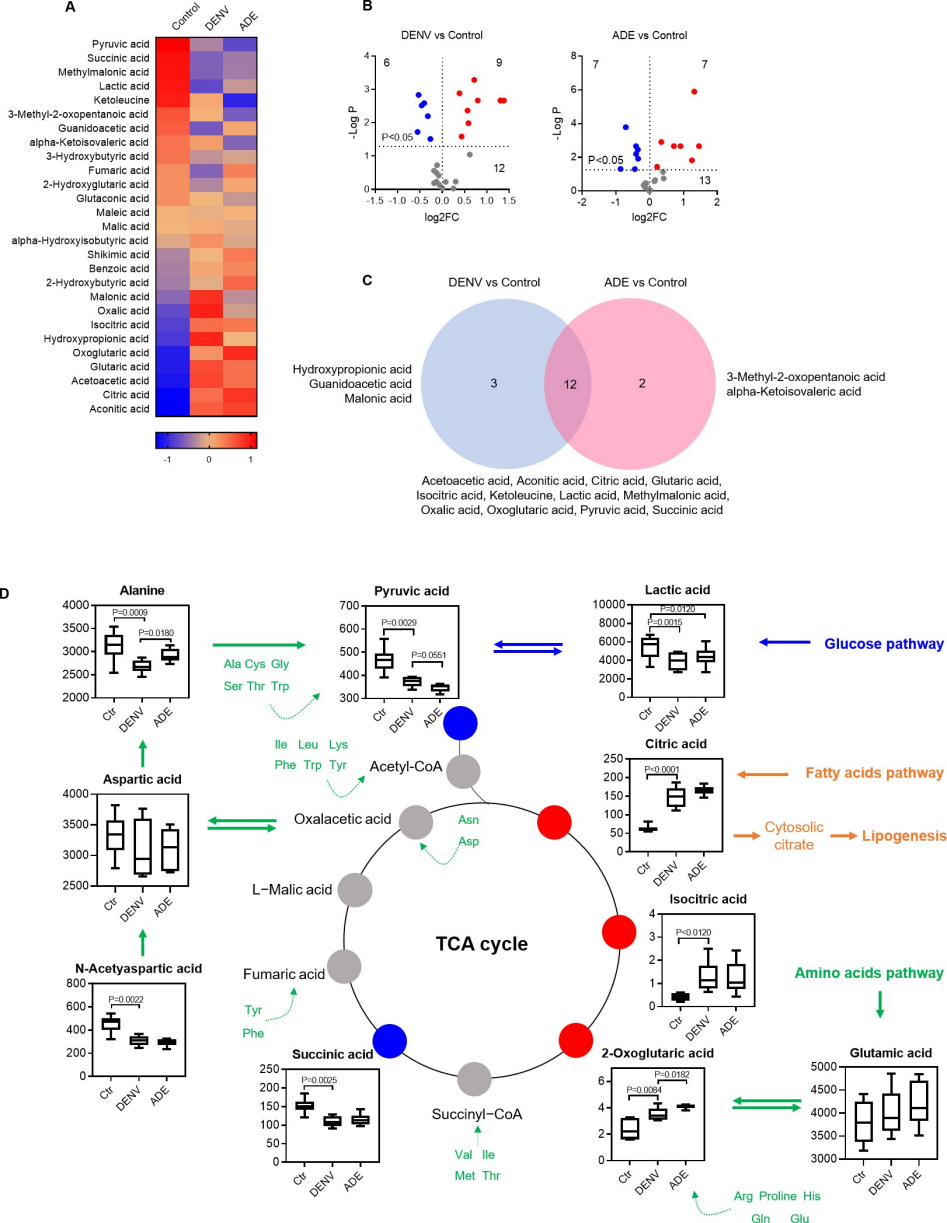
Dengue virus infection significantly changes cellular organic acid contents. **A:** heatmap of organic acid variants. The mean Z-score of sextuplicate in each group was shown. **B**: display of organic acid variants by DENV infection only or ADE infection in comparison with non-viral infection. The mean density of sextuplicate of each variant was shown. Three overlaid dots were shown in the left panel as aconitic acid, citric acid, and isocitric acid had a similar degree of increase. Blue dot: significantly decreased variants; red dot: significantly increased variants. **C**: distribution of significant organic acid variants under different infection conditions. **D**: organic acids bridge tricarboxylic acid cycle and metabolic pathways.

Intracellular organic acids are metabolic intermediates of various metabolic substrates (carbohydrates, fatty acids, and amino acids). DENV infection produced 27 variants of organic acids, 17 of them reached significant (FC >2, P<0.05), including 8 decreased and 9 increased variants (**Figs 7A and S7)**. DENV only infection induced significant decreases in 6 and increases in 9 variants (including hydroxypropionic acid, glutaric acid, aconitic acid, acetoacetic acid, citric acid, isocitric acid, oxalic acid, oxoglutaric acid, and malonic acid), three of them (aconitic acid, citric acid, and isocitric acid) had a similar degree of increase; whereas, ADE infection resulted in significant decreases in 7 and increases in 7 variants (**Fig 7B**). Among the significantly changed variants, 3 (hydroxypropionic acid, malonic acid, and guanidoacetic acid) were generated by DENV only infection, 2 variants (alpha-ketoisovaleric acid and 3-methyl-2-oxopentanoic acid) by ADE infection, and 12 (acetoacetic acid, aconitic acid, citric acid, glutaric acid, isocitric acid, ketoleucine, lactic acid, methylmalonic acid, oxalic acid, 2-oxoglutaric acid, pyruvic acid, succinic acid) were shared by both DENV only and ADE infections (**Fig 7C**).

In particular, the organic acids of pyruvate, citrate, isocitrate, 2-oxoglutarate, and succinate are the key components of tricarboxylic acid (TCA) cycle, which serves as the exchange hub of metabolic intermediates from metabolic pathways of glucose, fatty acids, and amino acids (**Fig 7D**). In addition, 3-methyl-2-oxobutanoic acid, 3-methyl-2-oxopentanoic acid, 4-methyl-2-oxopentanoate, acetoacetate, and methylmalonate are the products of branched-chain amino acids (valine, leucine, and isoleucine) degradation, mirroring the active amino acid catabolism during DENV viral infection as shown in **Fig 3**.

### Other metabolite variants by dengue virus infection

**Fig 8:**
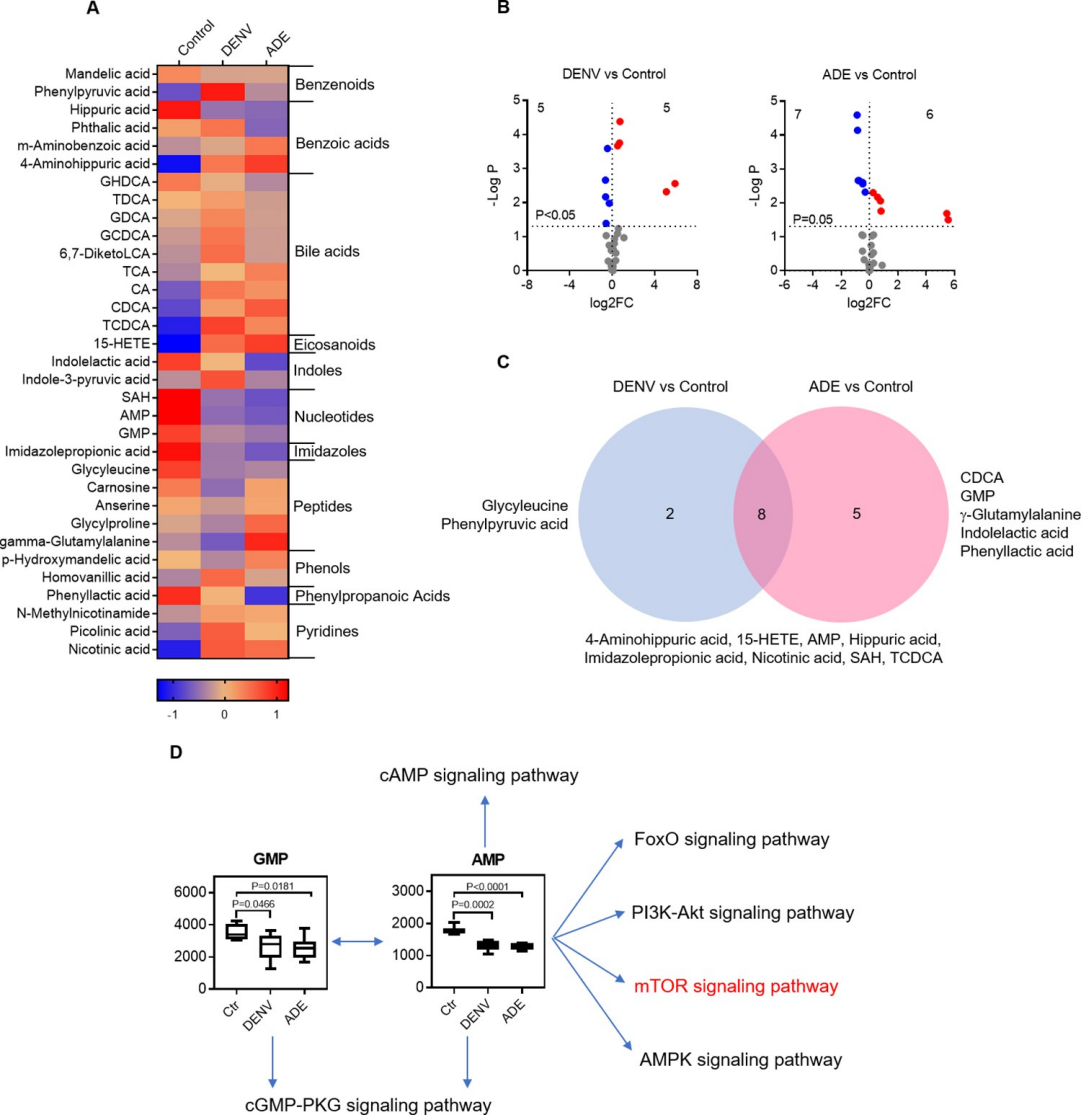
Other metabolite variants produced by dengue virus infection. **A:** heatmap of differentially expressed other metabolites covering benzenoids, benzoic acids, bile acids, eicosanoids, imidazoles, indoles, nucleotides, peptides, phenols, phenylpropanoic acids, and pyridines. The mean Z-score of sextuplicate in each group was shown. **B**: display of other metabolite variants by DENV infection only or ADE infection in comparison with non-viral infection. The mean density of sextuplicate of each variant was shown. Blue dot: significantly decreased variants; red dot: significantly increased variants. **C**: distribution of significant other metabolite variants under different infection conditions. **D**: purine nucleoside monophosphates impact multiple anabolic signaling pathways. Ctr: non-viral infection control.

The other metabolite variants occupied 16.35 of the all detected metabolite variants (**Fig 2A**). They were benzenoids, benzoic acids, bile acids, eicosanoids, imidazoles, indoles, nucleotides, peptides, phenols, phenylpropanoic acids, and pyridines. DENV infection produced 33 variants of other metabolites, 15 of them reached significant (FC>2, P<0.05), including 8 decreased and 7 increased variants. (**Figs 8A and S8)**. DENV only infection induced significant decreases in 5 and increases in 5 variants, whereas, ADE infection resulted in significant decreases in 7 and increases in 6 variants (**Fig 8B**). Among the significantly changed variants, 2 variants (glycyleucine and phenylpyruvic acid) were specific to DENV only infection, 5 variants (CDCA, GMP, γ-Glutamylalanine, indolelactic acid, and phenyllactic acid) specific to ADE infection, and 8 were shared by both non-ADE and ADE infections (**Fig 8C**).

Remarkably, the changes of AMP and GMP contents have physiological significance, they are one of the cellular energy statuses as well as signaling molecules. Their reduction in response to non-ADE or ADE DENV infection indicates a higher cellular energy status and alternations of several signaling pathways that govern many cellular physiological events such as metabolism, protein synthesis, secretion, calcium homeostasis, and gene transcription. For example, the low cellular level of AMP, i.e., a high ATP/AMP ratio, inhibits AMPK signaling but activates PI3K/AKT signaling pathways, which collectively activate mTOR signaling pathway to promote protein synthesis (**Fig 8D**).

### Differentially expressed metabolites of dengue virus infection

**Fig 9:**
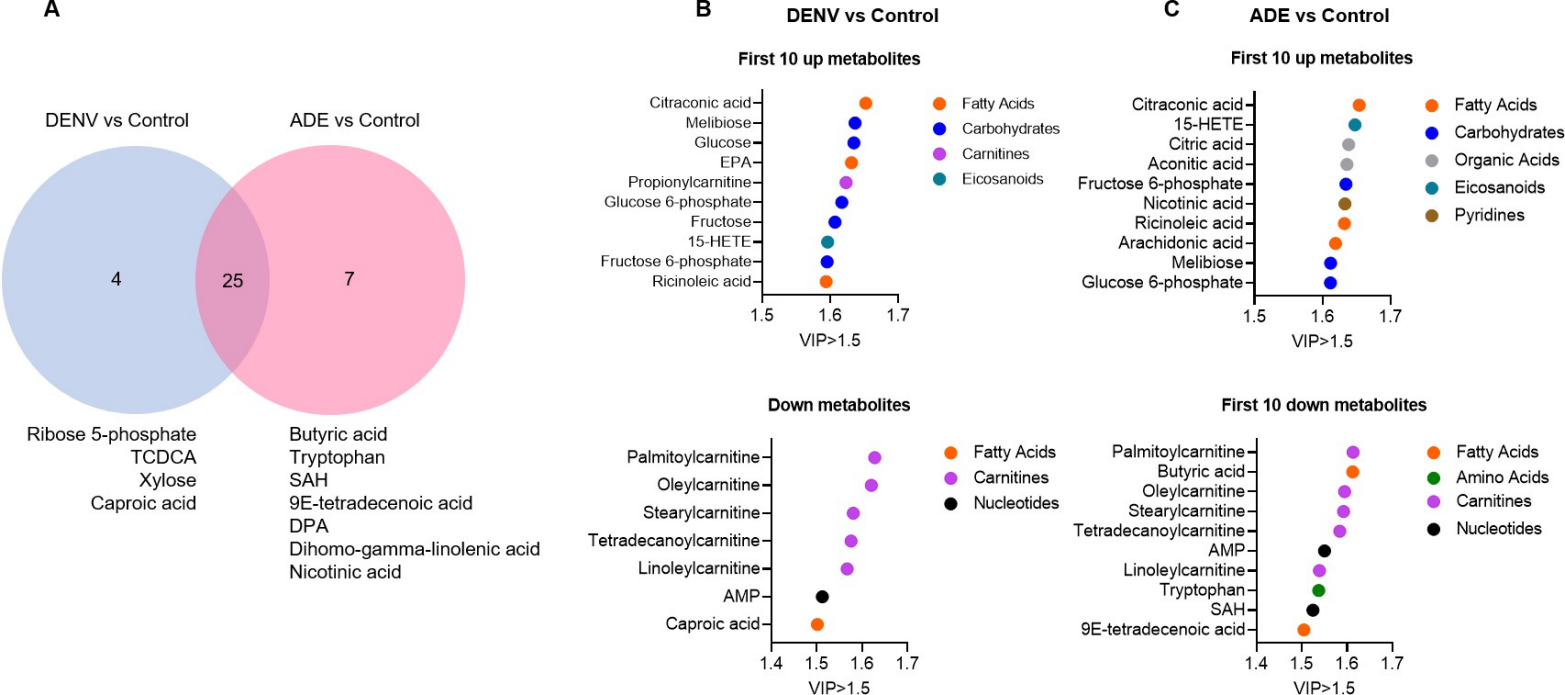
Differentially expressed metabolites of dengue virus infection. **A:** the association of differentially expressed metabolites with different infection conditions. **B**: top differentially expressed metabolites in DENV only infection. **C**: top differentially expressed metabolites in ADE infection.

In order to identify the differential metabolites with diagnostic significance, the variants with p<0.05 in single-dimensional tests and variable projection importance (VIP) >1.5 in multidimensional statistics were selected as differentially expressed metabolites. According to this statistic standard, totally 36 variants were differentially expressed metabolites in DENV infection, 11 of them were decreased and 25 were increased (**S2 Table**). To distinguish non-ADE from ADE infection, the decrease in caproic acid and increases in ribose 5-phosphate, TCDCA, and xylose were specifically differentially expressed metabolites in DENV only infection. Whereas, the decreases in butyric acid, SAH, 9E-tetradecenoic acid and the increases in nicotinic acid, dihomo-gamma-linolenic acid and DPA were specifically differentially expressed metabolites in ADE infection (**Fig 9A**). The elevated metabolic markers were mainly fatty acids and carbohydrates, while the reduced metabolic markers were mainly carnitines (**Fig 9B** and **9C**).

## Discussion

Although viruses differ greatly between species and category, they are obligate intracellular pathogens and must depend on metabolic processes of host cells for reproduction. Our metabolomic study provided several lines of evidence for the anabolic nature of dengue virus infected monocytes/macrophages.

Firstly, the overall exhaustion of cellular amino acids suggested an active protein biosynthesis. Amino acids are consisted of 9 essential, 7 conditionally non-essential, and 4 non-essential amino acids. They are the building blocks of protein and polypeptide biosynthesis and one of the substrates for energy generation. In addition, they are key precursors for syntheses of hormones and low-molecular weight nitrogenous substances such as purines, pyrimidines, coenzymes, and the biogenic amines [41], and each of them is biologically important. Amino acids are also the cell signaling molecules, and their changes in concentration are timely sensed by mTOR complex, which is the checkpoint molecule of the anabolism signaling pathway [42,43]. The global consumption of cellular amino acids prompts biosynthesis of aminoacyl-tRNA that supports protein anabolism of DENV infection. Importantly, degradation of the essential amino acid tryptophan produces immunosuppressive intermediates, such as kynurenine, 3-hydroxykynurenine, 3-hydroxyanthranilic acid, and quinolinic acid, facilitating the immune escape of viral infections [23,44,45].

Secondly, the increase in levels of cellular fatty acids indicated an active lipid biosynthesis. Fatty acids play a wide range of physiological roles [46]. They are not only one of the major sources of cellular fuel and energy storage, but also the precursors of hormones and lipids to participate in biological signaling pathways. Of particular importance is that fatty acids are the constituents of plasma membrane [18,21,47,48], making them necessary for replication cycle of dengue virus. Pharmacologically targeting host lipid synthesis can inhibit viral replication [49]. Remarkably, the polyunsaturated fatty acids contributed over 60% of the significant variants of fatty acids during DENV only and ADE infections, and most of them are involved in the pathway of unsaturated fatty acids biosynthesis to meet the demands of the active biosynthesis of viral envelope. In addition, carnitine is a non-essential amino acid and a quaternary ammonium compound. Its fatty acid ester acylcarnitine plays a key role in lipid metabolism and transfer cytosolic fatty acids into mitochondria by carnitine palmitoyltransferase 1, which is the rate-limiting step of fatty acid β-oxidation [50]. Significant decreases in short-, medium- and long-chain fatty acid-esterified carnitines indicate the repression of fatty acid catabolism, leading to the accumulation of cellular fatty acids. The importance of lipid biosynthesis in maintenance of dengue viral replication has also been highlighted in mosquito vector cells [51].

Thirdly, the general increase in levels of cellular carbohydrates inferred their active metabolism. Carbohydrates are catabolized via glycolysis and pentose phosphorylation pathways (PPP). The aerobic glycolysis of carbohydrates produces energy to meet the need of anabolism [52] and has been significantly involved in dengue virus infection [53]. Through the PPP pathway, the breakdown of carbohydrates produces precursors necessary for the biosynthesis of nucleotides, amino acids, one-carbon units, reducing molecules, and antioxidants [54]. This process is pivotal in anabolism and serves as a metabolic biomarker for viral pathogenesis [55]. However, the role of the pentose phosphate pathway varies with viral species, probably due to their difference in sensitivity to oxidative stress; for example, it promotes influenza virus but inhibits DENV and SARS-CoV-2 viral replications [56–58].

Finally, dengue viral infection dominantly activates metabolically anabolic pathways. In addition to the above-described glycolysis and PPP pathways, which provide energy, building blocks and antioxidants for anabolism, other activated metabolic pathways include the pathways for amino acid catabolism, aminoacyl-tRNA biosynthesis, fatty acid biosynthesis, and tricarboxylic acid (TCA) cycle, which is an essential metabolic network that connects aerobic metabolism of carbohydrate, fat, and protein (amino acid) [59]. The dynamics of the metabolic intermediates of TCA cycle reflect fluctuations of intracellular metabolic pathways during dengue virus infection.

The anabolic profile of the active DENV-infected macrophages has significance of immunometabolism, a concept that was proposed in early 1910s, based on the observation that the immune activation of leukocytes is coupled with degradation of 6-carbon glucose to 3-carbon lactate [60]. The current immunometabolic concept has been advanced to state the intersection between immunology and biochemistry by which metabolic pathways regulate immune cell growth, differentiation and function [61]. Pathogen infection triggers an anabolic cytokine storm response through Toll-like receptors (TLR)-mediated immune activation and simultaneously reprograms metabolism by activating glycolysis and pentose phosphate pathways, which provide immune cytokine storm with energy and building materials for biosynthesis of nucleic acids and proteins [62]. Distinct from bacterial infection, viral infection relies on the activation of anabolic pathways in host cells for viral replications. Thus, the anabolic feature of macrophages integrates the viral production and immune activation against viral infection.

Usually, the significant metabolic variants have clinical significance of diagnosis and prediction of outcomes. Although the ex vivo study is not suitable to explore clinically related biomarkers, based on the statistical standard of p<0.05 and VIP >1.5, 36 variants from carbohydrate, fat, and carnitine metabolism met the standard as differentially expressed metabolites, and 11 variants displayed between the non-ADE from ADE infection of dengue virus. However, their diagnostic and disease progression predictive significance needs to be validated in future clinical studies.

The disturbed metabolic profile of monocyte/macrophage by DENV infection in the current study is mostly consistent with previous studies on serum of dengue fever patients and humanized mice model of dengue infection [18,20–22], which displayed an overall decrease in levels of amino acids and increase in levels of fatty acids include polyunsaturated fatty acids. These common metabolic changes in *ex vivo* and *in vivo* settings may be necessary to meet the metabolic demands of viral replication and host defense response. However, the exceptions were that DNEV infection of monocytes/macrophages induced a global reduction of acylcarnitines rather than the increase in serum metabolomics of dengue fever patients, and that the patient serum studies consistently showed active phospholipid catabolism, which did not show up in the current study on monocyte/macrophages. The metabolic dissimilarity may be attributed to the fact that changes in serum contents refract the orchestrated response of multiple organ systems including the immune system and the central metabolic organs liver and muscle. These *in vivo* studies have associated the metabolic changes with vital organ dysfunctions, disease development phases and severity, and were restorable upon disease recovery.

In summary, the current study disclosed an anabolism signature of dengue viral infection in macrophages, highlighting the integration of immune system and metabolism during the process of viral infection. However, our study met limitations to validate the findings via *in vivo* experiments due to the lack of suitable animal models of DENV-ADE infection in immunocompetent mice. In addition, patients with different serotypes of DENV infection indeed displayed different metabolic changes [24], thus the metabolic profiles of immune cells by different serotypes of DENV infection should be clarified in future studies.

## Supporting information

S1 Fig Multivariate Control Chart. The PC1 results indicated overall stable of the QC. The horizontal coordinate is the order in which all samples were tested, and the vertical coordinate is the PC1 scores. Each black dot in the graph represents a sample, and yellow dots represent quality control samples.
(TIF)

S2 Fig The proportion of various metabolites by metabolite class statistic analysis. (TIF)

S3 Fig DENV infection significantly reduces intracellular amino acid contents. Mean ± SEM of sextuplicate was shown.
(TIF)

S4 Fig DENV infection increases intracellular carbohydrate levels. Mean ± SEM of sextuplicate was shown.
(TIF)

S5 Fig DENV infection significantly changes intracellular fatty acid levels. Mean ± SEM of sextuplicate was shown.
(TIF)

S6 Fig DENV infection significantly reduces intracellular acylcarnitine levels. Mean ± SEM of sextuplicate was shown.
(TIF)

S7 Fig DENV infection significantly changes intracellular organic acid contents. Mean ± SEM of sextuplicate was shown.
(TIF)

S8 Fig DENV infection induces other intracellular metabolite contents. Mean ± SEM of sextuplicate was shown.
(TIF)

S1 Table Overall metabolite variants across the treatments. (PDF)

S2 Table Overall differentially expressed metabolites. (PDF)
