# Supplementary figures and images for: Metabolomic landscape of macrophage discloses an anabolic signature of dengue virus infection and antibody-dependent enhancement of viral infection
Source: PLoS Negl Trop Dis. 2024 Feb 2;18(2):e0011923. doi: 10.1371/journal.pntd.0011923 (PMC10866464; doi:10.1371/journal.pntd.0011923)

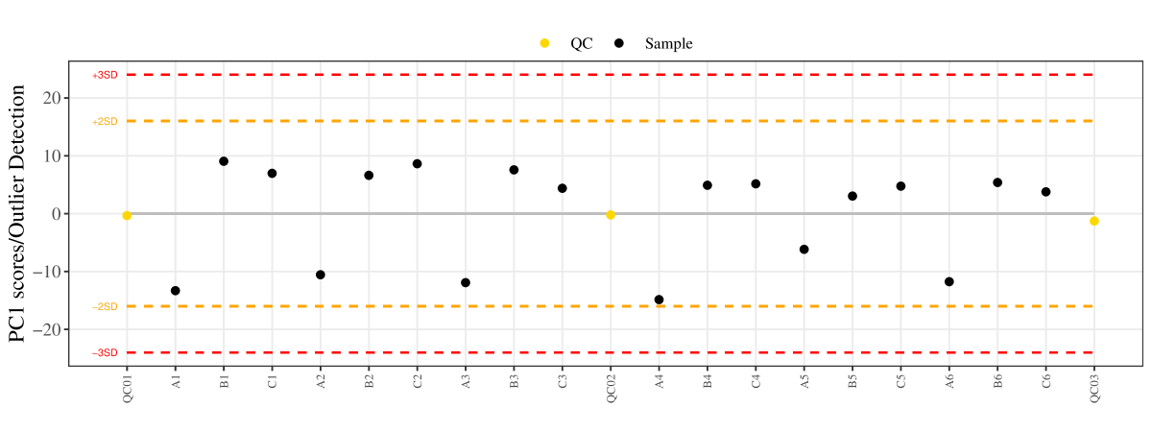

Supplement: S1 Fig — The PC1 results indicated overall stable of the QC. The horizontal coordinate is the order in which all samples were tested, and the vertical coordinate is the PC1 scores. Each black dot in the graph represents a sample, and yellow dots represent quality control samples. (TIF) [file pntd.0011923.s001.tif]

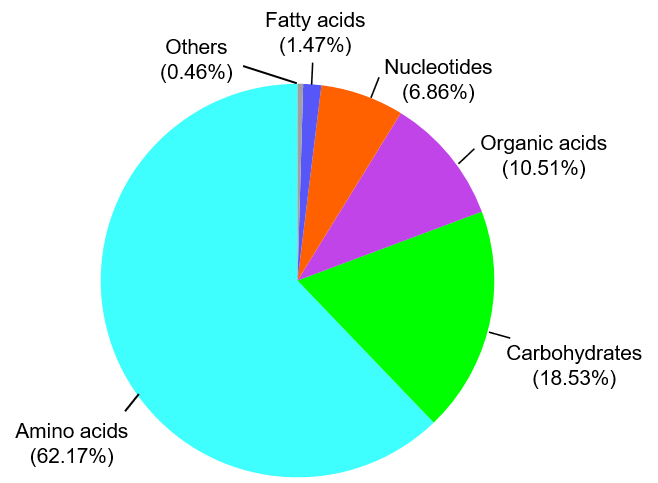

Supplement: S2 Fig — (TIF) [file pntd.0011923.s002.tif]

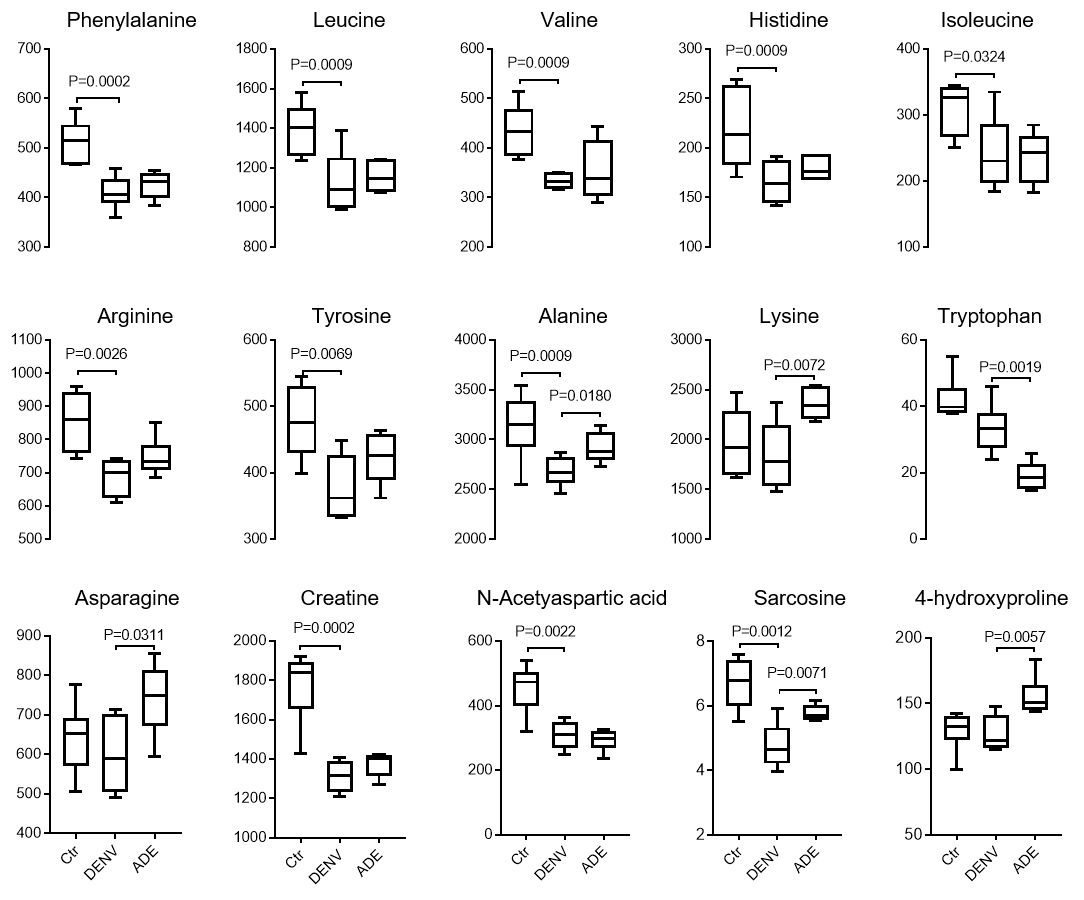

Supplement: S3 Fig — Mean ± SEM of sextuplicate was shown. (TIF) [file pntd.0011923.s003.tif]

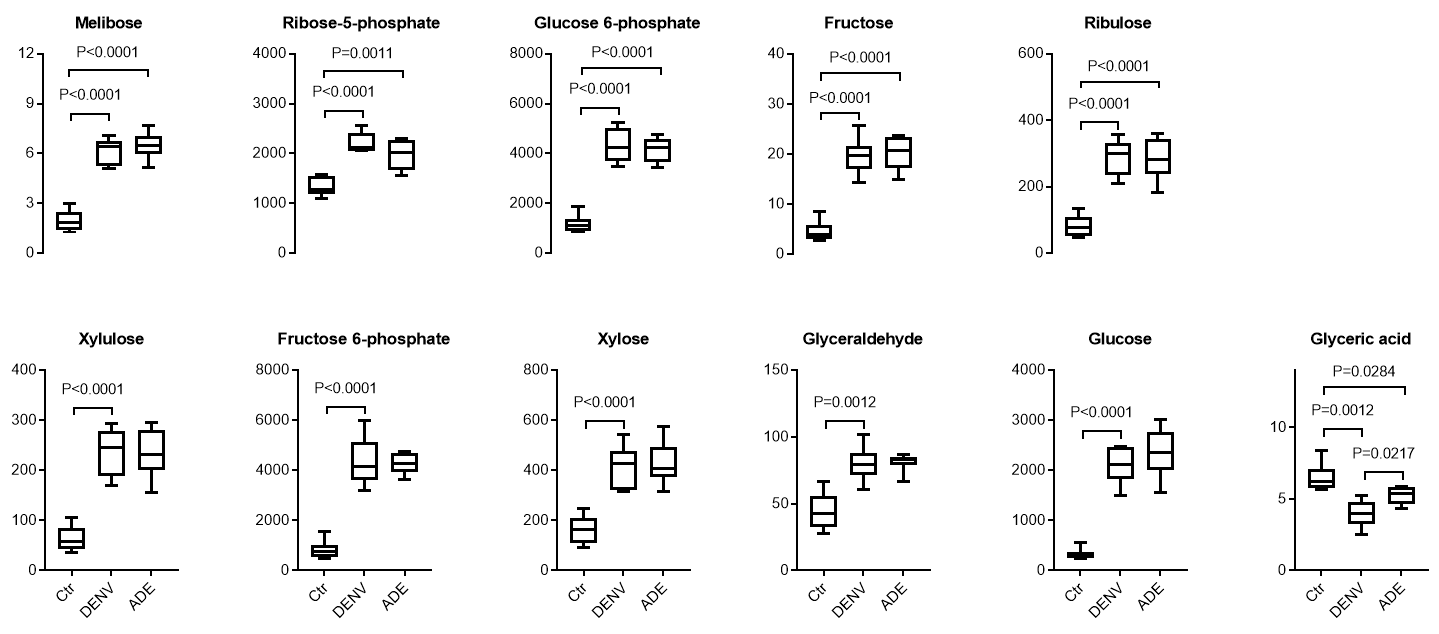

Supplement: S4 Fig — Mean ± SEM of sextuplicate was shown. (TIF) [file pntd.0011923.s004.tif]

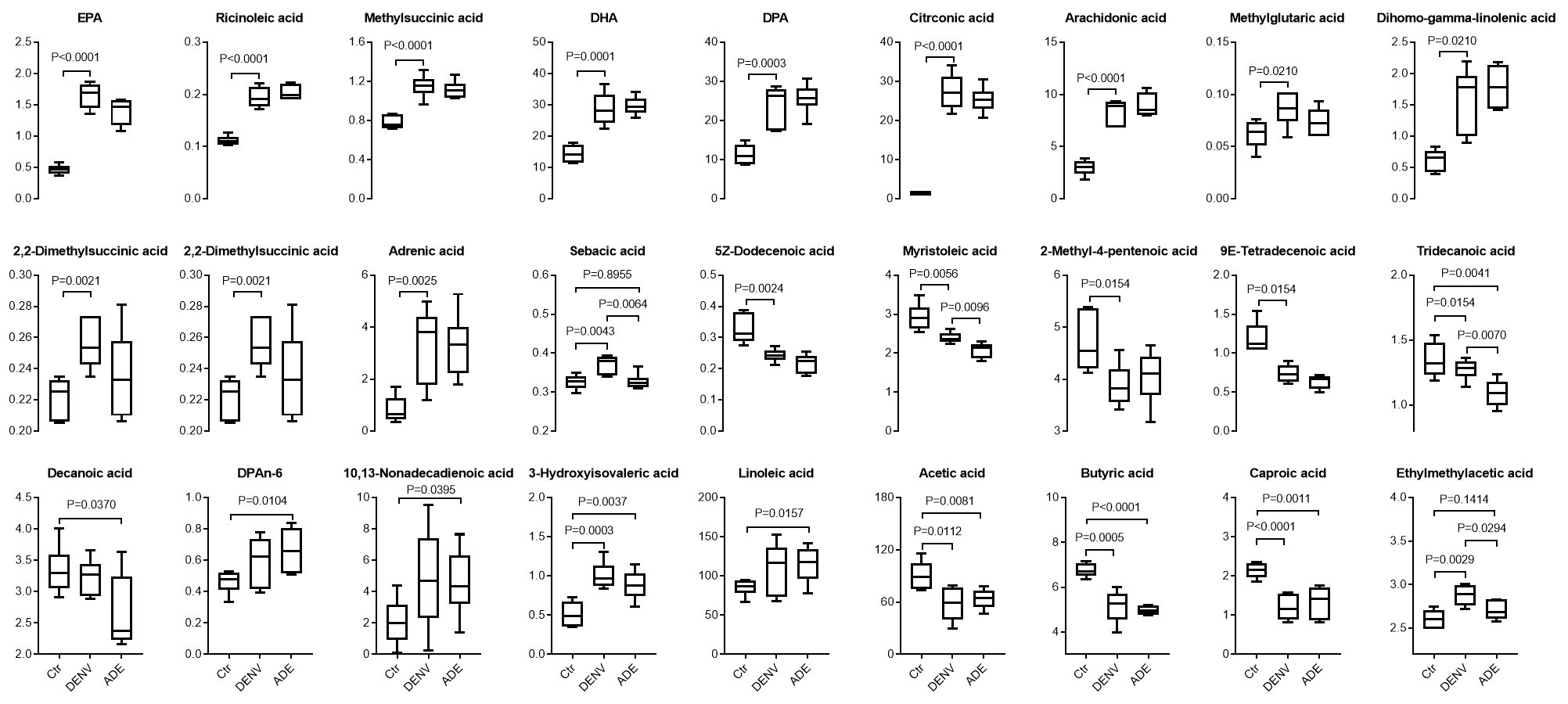

Supplement: S5 Fig — Mean ± SEM of sextuplicate was shown. (TIF) [file pntd.0011923.s005.tif]

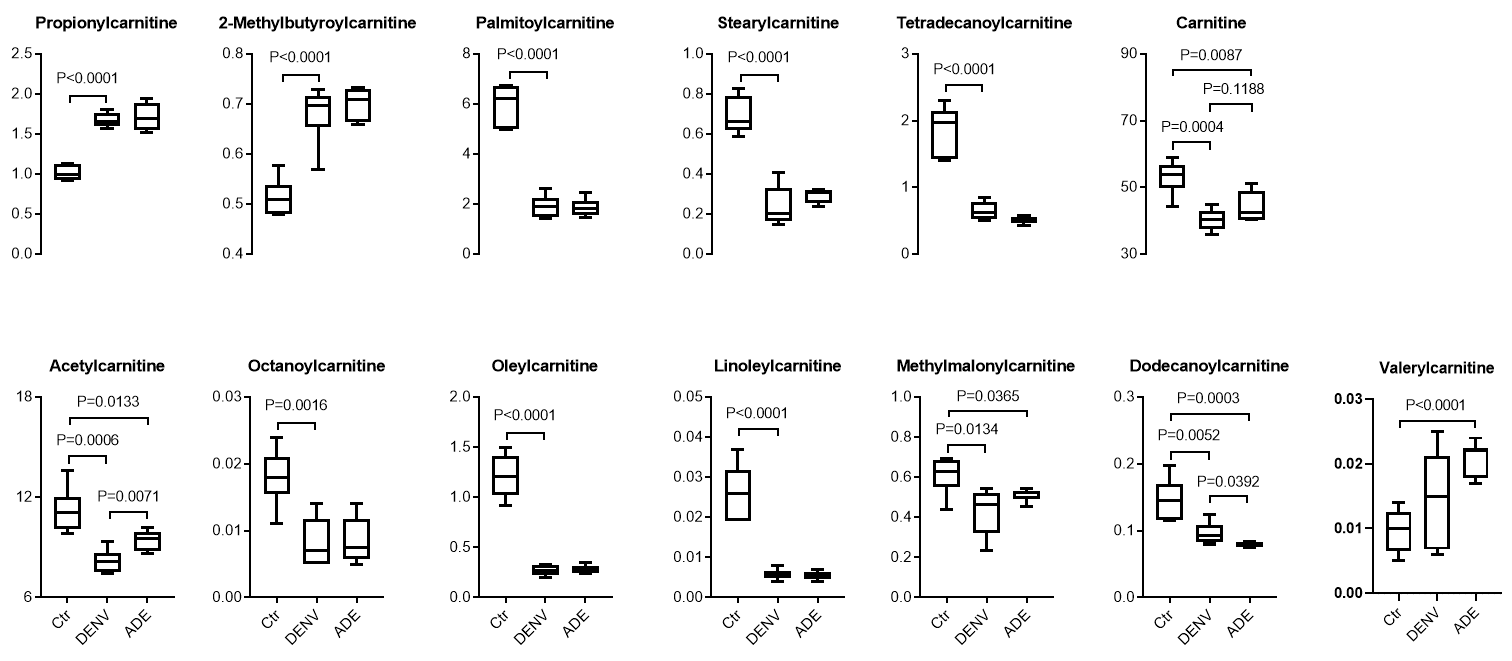

Supplement: S6 Fig — Mean ± SEM of sextuplicate was shown. (TIF) [file pntd.0011923.s006.tif]

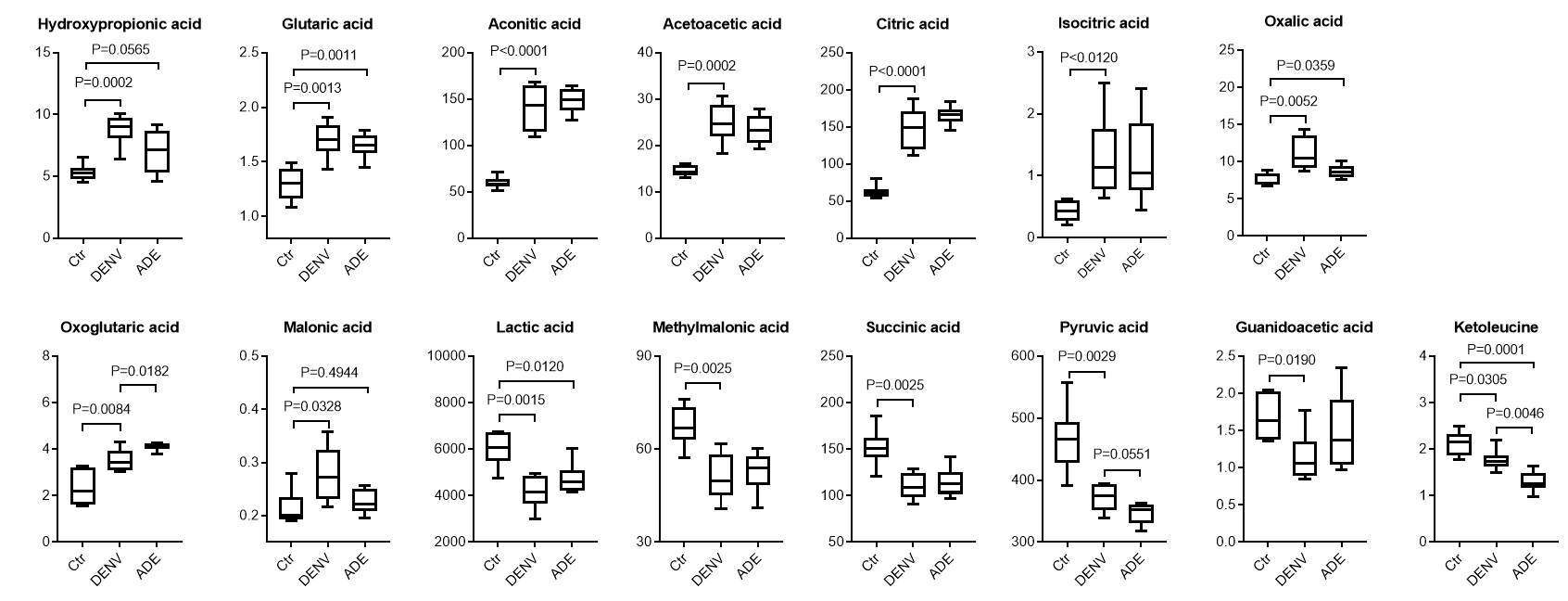

Supplement: S7 Fig — Mean ± SEM of sextuplicate was shown. (TIF) [file pntd.0011923.s007.tif]

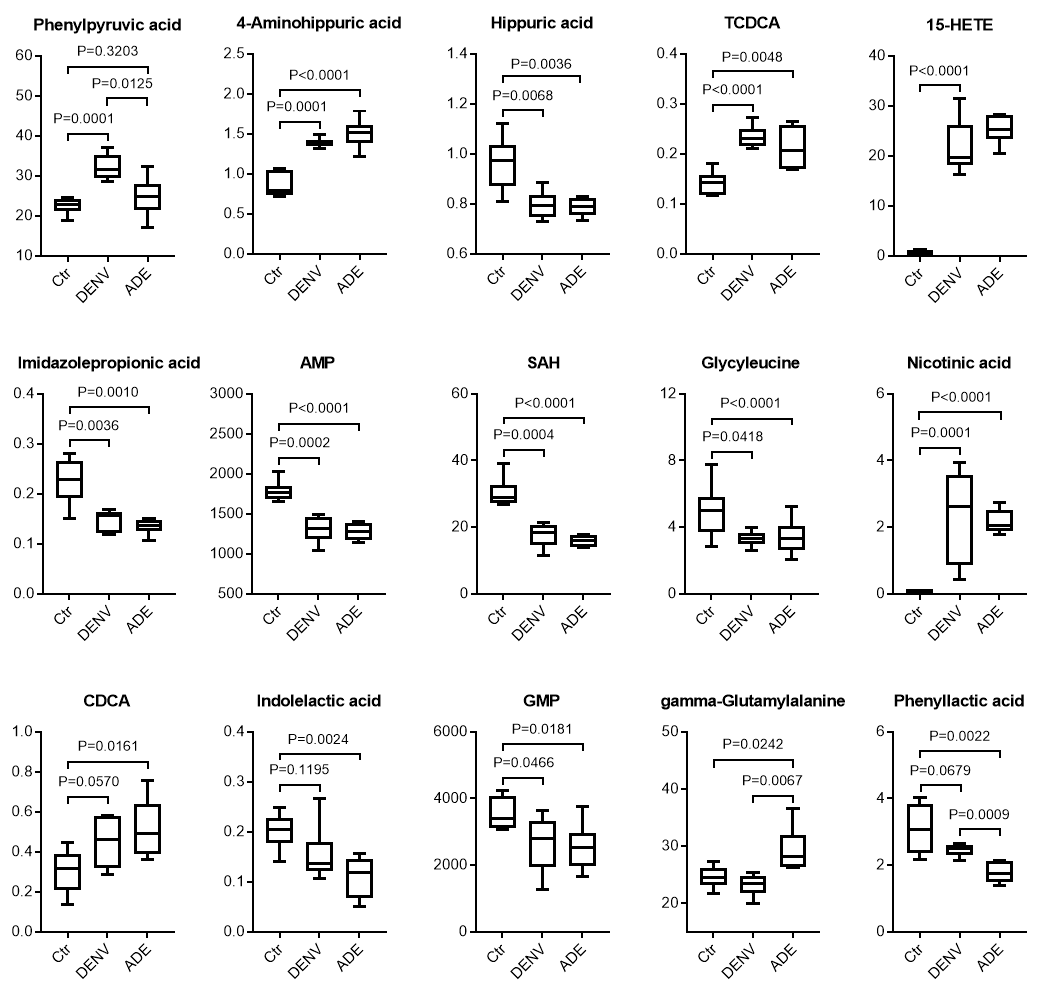

Supplement: S8 Fig — Mean ± SEM of sextuplicate was shown. (TIF) [file pntd.0011923.s008.tif]
