# Supplementary material for: Metabolomic landscape of macrophage discloses an anabolic signature of dengue virus infection and antibody-dependent enhancement of viral infection
Source: PLoS Negl Trop Dis. 2024 Feb 2;18(2):e0011923. doi: 10.1371/journal.pntd.0011923 (PMC10866464; doi:10.1371/journal.pntd.0011923)
Supplement: S1 Table — (PDF) [file pntd.0011923.s009.pdf]

S1 Table : Overall meatbolite variants across the treatments

				Cell only						DENV-2 infection							DENV-2 + anti-DENV-3 serium					Quality control		
Class	HMDB	KEGG	Metabolite	A1	A2	A3	A4	A5	A6	B1	B2	B3	B4	B5	B6	C1	C2	C3	C4	C5	C6	QC01	QC02	QC03
Amino Acids	HMDB0000182	C00047	Lysine	2476.576	2063.601	1772.536	1620.201	1667.742	2201.827	2054.23	2371.523	1479.112	1965.865	1569.227	1601.302	2518.187	2176.746	2544.563	2287.076	2240.043	2396.878	1844.364	1665.753	1584.993
Amino Acids	HMDB0000177	C00135	Histidine	229.933	269.193	189.301	170.608	197.107	259.81	141.5	191.923	146.949	173.659	154.996	184.749	168.647	176.913	169.116	176.293	192.367	192.084	178.155	184.456	155.546
Amino Acids	HMDB0000517	C00062	Arginine	960.288	929.807	825.637	741.808	772.424	895.173	743.844	611.102	689.922	710.275	731.016	635.467	754.801	744.598	849.992	722.998	723.529	685.07	819.29	786.01	783.68
Amino Acids	HMDB0000641	C00064	Glutamine	492.177	453.165	301.863	311.053	316.405	403.888	334.671	337.322	322.594	625.607	451.414	324.58	386.172	338.27	424.924	406.976	435.074	379.087	415.392	377.632	393.431
Amino Acids	HMDB0000148	C00025	Glutamic acid	4192.44	3637.923	4412.766	3184.901	3435.389	3948.306	4283.659	4089.64	3665.646	3436.44	4856.656	3698.274	3969.763	4654.478	4250.98	3930.212	4851.02	3509.591	4425.538	4539.566	4293.743
Amino Acids	HMDB0000056	C00099	beta-Alanine	779.854	727.74	687.899	736.496	497.946	608.439	705.02	568.812	538.676	665.05	739.33	643.173	656.384	637.977	708.092	643.357	745.852	745.435	699.113	649.248	677.795
Amino Acids	HMDB0000161	C00041	Alanine	3312.231	3540.486	3072.547	3144.633	2543.218	3157.142	2702.105	2630.498	2621.814	2789.53	2867.987	2458.559	2730.138	2834.623	3143.324	2857.306	2900.513	3032.011	3065.707	2874.246	3205.683
Amino Acids	HMDB0000112	C00334	GABA	17.424	17.93	17.271	18.373	13.235	15.968	13.463	13.644	12.118	14.515	15.278	11.591	11.274	12.883	14.212	13.401	16.525	13.122	13.93	14.052	17.686
Amino Acids	HMDB0000187	C00065	Serine	1334.314	1281.946	1024.339	1107.579	1053.82	1303.711	951.338	980.988	918.984	1134.981	1162.665	863.818	1026.57	1009.015	1019.884	1078.089	1143.959	1037.381	1132.627	1049.95	1049.493
Amino Acids	HMDB0000167	C00188	Threonine	655.435	902.891	655.354	765.294	792.128	843.296	494.636	509.262	829.451	774.892	574.193	488.796	488.704	527.112	592.019	801.354	642.046	615.365	641.802	588.198	686.264
Amino Acids	HMDB0000064	C00300	Creatine	1857.649	1883.82	1820.097	1921.462	1429.315	1728.993	1361.329	1208.891	1267.047	1405.171	1383.333	1240.647	1325.705	1270.857	1415.84	1401.661	1402.395	1423.813	1587.087	1487.264	1590.011
Amino Acids	HMDB0001901	C02378	Aminocaproic acid	1.949	1.072	1.6	1.734	1.662	1.977	1.427	1.825	1.496	1.771	2.527	1.658	1.521	0.871	1.319	2.106	2.146	2.107	2.116	1.088	1.979
Amino Acids	HMDB0002108	NA	Methylcysteine	17.275	14.213	14.506	13.262	11.76	14.5	18.562	16.756	13.63	12.871	16.09	14.695	17.382	16.754	18.093	16.252	19.169	15.181	13.973	15.394	15.314
Amino Acids	HMDB0000158	C00082	Tyrosine	522.734	545.455	443.719	446.975	398.995	502.905	370.198	353.205	335.473	416.697	448.014	332.519	361.793	411.322	441.568	454.044	463.236	401.642	426.177	421.494	446.322
Amino Acids	HMDB0000168	C00152	Asparagine	777.651	649.924	655.464	590.718	507.207	662.807	696.653	713.273	489.711	508.873	663.495	517.033	698.32	798.71	784.064	593.191	854.234	711.62	753.865	701.014	738.084
Amino Acids	HMDB0000159	C00079	Phenylalanine	532.571	579.06	469.093	515.674	466.671	516.019	405.3	359.46	425.569	403.456	458.896	406.603	383.135	409.113	453.765	444.997	440.255	424.958	459.129	450.818	464.904
Amino Acids	HMDB0000684	C00328	Kynurenine	23.166	17.51	14.099	17.388	14.015	16.125	6.208	9.855	8.208	14.555	13.838	12.621	10.865	10.235	15.168	14.937	16.105	15.245	13.859	16.378	14.568
Amino Acids	HMDB0000191	C00049	Aspartic acid	3485.237	3312.611	3371.95	3822.242	2791.974	3182.441	2653.8	2699.132	2930.905	3538.696	3766.772	2959.743	2726.282	2748.622	3129.67	3130.092	3503.244	3399.819	2982.788	3585.438	3366.062
Amino Acids	HMDB0000510	C00956	Aminoadipic acid	36.664	59.15	54.381	63.582	60.244	50.334	38.331	55.961	83.628	76.986	61.156	42.6	33.396	39.779	58.565	84.559	74.04	49.989	49.7	53.192	44.36
Amino Acids	HMDB0000766	NA	N-Acetyalanine	91.749	114.896	99.933	126.852	46.982	77.758	53.499	69.37	69.996	80.124	66.82	75.739	77.755	69.959	106.715	101.454	89.756	63.833	95.76	83.446	89.03
Amino Acids	HMDB0000812	C01042	N-Acetyaspartic acid	540.544	456.6	490.901	491.369	321.362	424.853	322.586	278.08	248.423	297.696	364.09	343.85	297.246	236.325	299.778	279.264	318.502	325.592	321.981	346.697	350.256
Amino Acids	HMDB0002372	NA	N-Phenylacetylphenylalanine	0.112	0.081	0.0071	0.0071	0.08	0.074	0.113	0.084	0.071	0.092	0.091	0.08	0.085	0.0071	0.095	0.09	0.078	0.094	0.088	0.075	0.089
Amino Acids	HMDB0000123	C00037	Glycine	6783.053	6240.343	5944.089	6367.048	5143.421	5893.048	5801.154	5701.926	5022.673	5532.041	6183.553	5447.092	5062.778	5424.723	5866.436	5611.118	6147.792	5367.222	6053.265	5934.921	5814.583
Amino Acids	HMDB0000904	C00327	Citrulline	1.071	1.819	1.385	0.388	4.729	0.331	2.562	1.71	0.883	1.364	0.568	1.233	2.43	1.09	1.13	1.491	0.533	1.414	1.274	1.429	1.247
Amino Acids	HMDB0000452	C02356	alpha-Aminobutyric acid	85.312	45.294	50.978	52.397	32.848	32.747	54.577	63.046	26.102	36.138	47.068	42.342	48.233	73.29	64.034	41.77	57.583	59.869	67.529	55.551	50.875
Amino Acids	HMDB0000162	C00148	Proline	1154.492	989.941	1111.664	900.364	859.243	1070.562	1003.865	1073.851	826.383	862.906	1195.988	945.22	1024.545	1224.938	1233.311	1025.104	1365.415	986.767	1171.887	1179.707	1164.332
Amino Acids	HMDB0000532	NA	Acetylglycine	19.789	13.368	17.164	15.108	9.348	11.582	11.51	12.533	7.499	9.291	10.652	10.589	9.053	13.944	14.669	8.238	12.161	13.979	15.443	12.801	12.775
Amino Acids	HMDB0000716	C00408	Pipecolic acid	1.754	1.481	1.464	1.823	1.426	1.624	1.461	1.698	1.281	1.704	1.777	1.663	1.492	1.532	1.747	1.579	1.876	1.791	1.394	1.758	1.737
Amino Acids	HMDB0002931	NA	N-Acetylserine	293.391	203.03	226.799	200.305	132.398	150.199	190.52	235.11	116.564	174.324	230.429	238.088	189.033	283.539	266.448	166.922	264.601	273.445	256.637	204.628	222.412
Amino Acids	HMDB0006029	NA	N-Acetylglutamine	2.774	1.961	2.597	2.324	1.653	1.808	2.695	3.133	1.637	2.307	2.22	2.52	2.604	2.656	2.424	2.177	2.794	2.649	2.627	2.602	2.234
Amino Acids	HMDB0000883	C00183	Valine	513.755	454.079	463.897	412.481	377.108	390.182	330.774	316.269	320.751	347.226	350.891	332.852	289.233	442.718	402.441	339.579	336.32	312.262	484.916	332.039	331.631
Amino Acids	HMDB0000267	C01879	Pyroglutamic acid	13649.95	9491.35	11718.31	9438.571	6547.509	7634.767	11454.16	11200.36	7161.038	8454.508	10568.47	11556.58	10369.96	14912.43	12755.43	8546.518	11471.9	12335.77	13305.6	10124.27	10015.05
Amino Acids	HMDB0001149	C00430	5-Aminolevulinic acid	89.589	66.338	87.83	77.17	50.743	56.207	82.553	92.4	45.069	65.83	89.045	89.054	75.446	93.505	97.227	53.209	87.135	104.7	95.145	74.595	74.588
Amino Acids	HMDB0000696	C00073	Methionine	480.725	573.897	418.656	468.075	431.406	497.09	484.355	455.975	444.849	532.15	590.848	437.169	490.206	462.511	574.325	540.835	517.99	486.447	498.078	507.05	500.755
Amino Acids	HMDB0000172	C00407	Isoleucine	275.795	315.345	249.889	336.496	339.143	344.593	183.95	206.985	266.568	334.682	253.411	203.841	203.728	183.076	252.389	284.6	258.94	234.263	213.821	215.515	230.319
Amino Acids	HMDB0000687	C00123	Leucine	1420.756	1576.889	1235.656	1385.734	1279.394	1464.572	1026.364	1151.441	1006.537	1196.322	1389.363	991.218	1076.671	1091.392	1241.551	1156.375	1233.24	1132.831	1236.195	1211.965	1211.516
Amino Acids	HMDB0000929	C00078	Tryptophan	38.719	54.895	37.822	39.856	39.714	41.803	32.693	23.983	34.644	34.18	45.993	29.241	20.82	14.499	16.389	25.808	21.058	15.696	28.9	32.603	32.344
Amino Acids	HMDB0006344	C04148	Phenylacetylglutamine	0.002	0.01	0.003	0.004	0.005	0.002	0.002	0.003	0.072	0.002	0.011	0.015	0.001	0.001	0.006	0.009	0.013	1.00E-04	0.014	0.025	0.016
Amino Acids	HMDB0032055	C02997	N-Acetylhistidine	1.393	2.392	0.1071	2.327	1.502	1.684	1.368	2.542	1.12	0.1071	1.918	2.153	1.722	1.287	1.071	1.155	1.417	2.165	1.631	1.114	2.016
Amino Acids	HMDB0000001	C01152	1-Methylhistidine	12.122	8.315	7.341	7.091	5.726	5.91	7.796	7.586	4.983	6.52	5.885	6.177	9.563	7.929	9.062	6.825	7.833	6.044	7.208	6.649	7.343
Amino Acids	HMDB0000725	C01157	4-Hydroxyproline	142.013	129.571	130.028	139.606	99.874	135.277	138.816	114.45	116.985	125.65	147.418	116.654	151.295	145.526	157.506	144.003	149.432	183.576	142.322	132.273	141.361
Amino Acids	HMDB0000214	C00077	Ornithine	83.162	61.938	49.877	47.209	43.916	77.633	75.115	43.868	34.017	42.588	62.291	46.1	40.466	79.735	77.412	68.353	62.797	40.724	69.802	52.276	55.768
Amino Acids	HMDB0000192	C00491	Cystine	15.186	10.935	13.768	11.556	13.763	15.031	13.143	22.831	10.346	14.776	29.32	15.847	10.756	17.098	18.861	14.682	23.155	17.711	15.244	18.447	21.034
Amino Acids	HMDB0000271	C00213	Sarcosine	7.369	7.579	6.152	6.427	5.502	7.138	4.535	5.128	5.916	4.294	4.721	3.953	5.572	5.576	5.959	5.843	5.532	6.161	6.378	5.454	6.049
Amino Acids	HMDB0000092	C01026	Dimethylglycine	0.997	1.01	1.019	1.433	0.766	0.937	0.96	0.903	0.582	1.08	1.116	0.971	0.991	0.762	1.23	0.678	0.969	1.366	1.283	0.961	1.151
Amino Acids	HMDB0000719	C00263	Homoserine	4.54	3.364	3.172	4.928	3.388	4.921	2.78	3.348	2.457	3.62	3.752	2.946	3.061	2.328	3.114	4.209	5.71	4.597	3.401	3.161	4.213
Amino Acids	HMDB0000679	C02427	Homocitrulline	0.475	0.593	0.539	0.529	0.471	0.424	0.248	0.473	0.499	0.464	0.255	0.375	0.712	0.394	0.435	0.29	0.433	0.549	0.329	0.345	0.387
Amino Acids	HMDB0011745	C02712	N-Acetyl-L-methionine	7.239	19.656	14.516	16.351	10.252	14.592	16.259	19.989	13.426	15.209	4.177	11.963	12.886	24.589	18.129	19.059	14.777	10.702	13.799	19.685	17.496
Benzenoids	HMDB0000205	C00166	Phenylpyruvic acid	24.203	24.526	22.933	22.505	18.814	21.906	29.838	28.506	32.933	30.133	34.406	37.059	17.057	26.012	23.074	26.604	32.215	23.716	27.678	31.053	27.745
Benzenoids	HMDB0000703	C01984	Mandelic acid	0.081	0.035	0.016	0.023	0.102	0.092	0.028	0.094	0.056	0.021	0.062	0.008	0.041	0.027	0.022	0.049	0.076	0.055	0.083	0.082	0.066
Benzoic Acids	HMDB0001891	NA	m-Aminobenzoic acid	0.229	0.187	0.208	0.257	0.202	0.371	0.26	0.337	0.212	0.286	0.265	0.166	0.413	0.2	0.371	0.33	0.215	0.232	0.282	0.154	0.269
Benzoic Acids	HMDB0000714	C01586	Hippuric acid	1.119	1.008	0.889	1.009	0.811	0.941	0.817	0.753	0.73	0.795	0.794	0.887	0.83	0.823	0.795	0.762	0.732	0.786	0.746	0.829	0.825
Benzoic Acids	HMDB0001867	D06890	4-Aminohippuric acid	0.834	1.063	0.742	1.036	0.75	0.712	1.487	1.375	1.31	1.362	1.354	1.394	1.443	1.521	1.785	1.211	1.519	1.558	1.373	1.002	1.272
Benzoic Acids	HMDB0002107	C01606	Phthalic acid	0.25	0.276	0.341	0.3	0.341	0.317	0.234	0.404	0.405	0.245	0.43	0.221	0.231	0.322	0.205	0.216	0.312	0.216	0.316	0.321	0.376

Bile Acids	HMDB0000036	C05122	TCA	0.167	0.175	0.168	0.188	0.159	0.16	0.189	0.178	0.137	0.152	0.2	0.204	0.216	0.187	0.194	0.172	0.152	0.184	0.163	0.173	0.139
Bile Acids	HMDB0000896	C05463	TDCA	0.169	0.131	0.152	0.146	0.16	0.122	0.164	0.195	0.124	0.139	0.111	0.167	0.135	0.14	0.16	0.155	0.116	0.146	0.158	0.136	0.142
Bile Acids	HMDB0000951	C05465	TCDCA	0.181	0.151	0.117	0.134	0.119	0.15	0.272	0.211	0.226	0.218	0.243	0.234	0.264	0.211	0.254	0.169	0.2	0.17	0.141	0.186	0.172
Bile Acids	NA	NA	GHDCA	0.041	0.045	0.038	0.052	0.065	0.038	0.042	0.043	0.043	0.054	0.0038	0.042	0.066	0.043	0.0038	0.0038	0.038	0.044	0.042	0.039	0.045
Bile Acids	HMDB0000619	C00695	CA	0.107	0.096	0.0088	0.0088	0.092	0.092	0.094	0.094	0.104	0.096	0.115	0.091	0.102	0.09	0.091	0.099	0.088	0.093	0.094	0.097	0.093
Bile Acids	HMDB0000518	C02528	CDCA	0.23	0.305	0.137	0.444	0.372	0.324	0.396	0.284	0.58	0.582	0.528	0.332	0.4	0.596	0.359	0.456	0.529	0.759	0.465	0.445	0.314
Bile Acids	HMDB0000637	C05466	GCDCA	0.05	0.082	0.071	0.061	0.035	0.04	0.079	0.051	0.114	0.068	0.031	0.088	0.038	0.052	0.091	0.079	0.035	0.047	0.053	0.073	0.093
Bile Acids	HMDB0000631	C05464	GDCA	0.003	0.004	0.003	0.006	0.024	2.00E-04	0.007	0.01	0.008	0.004	0.022	0.007	2.00E-04	0.014	0.009	0.007	0.006	0.002	0.004	0.003	0.005
Bile Acids	NA	NA	6,7-DiketoLCA	6.758	7.59	7.745	7.36	7.957	8.585	7.301	7.611	7.933	9.337	9.076	7.893	6.945	7.705	7.792	7.85	8.599	7.468	7.274	8.378	8.634
Carbohydrates	HMDB0000663	C00818	Glucaric acid	1.144	0.956	1.161	1.252	0.787	1.362	1.061	1.201	0.711	1.615	1.278	1.12	0.837	0.692	1.108	0.909	1.346	1.695	0.938	1.69	1.191
Carbohydrates	HMDB0000139	C00258	Glyceric acid	8.375	6.675	5.663	5.785	6.161	6.297	4.639	5.252	2.489	4.116	3.789	3.542	4.701	5.021	5.869	4.344	5.736	5.838	5.282	5.165	6.332
Carbohydrates	HMDB0000230	C00270	N-Acetylneuraminic	1479.427	978.169	1157.279	1266.765	611.65	771.567	1078.987	1272.008	594.035	967.664	1149.79	1329.863	1010.264	1359.068	1616.538	984.843	1235.462	1631.029	1292.682	1052.644	1085.29
Carbohydrates	HMDB0000048	C05402	Melibiose	1.856	2.953	1.265	1.773	2.322	1.417	6.355	7.081	5.3	6.525	6.628	5.062	6.448	5.148	6.236	6.481	6.871	7.662	4.331	3.726	5.145
Carbohydrates	HMDB0000122	C00221	Glucose	300.33	549.739	260.816	247.809	263.117	300.517	1990.219	2479.684	1489.34	2467.978	2222.215	1931.947	1551.826	2670.497	2162.1	2165.144	3007.183	2528.871	1609.936	1611.557	1609.787
Carbohydrates	HMDB0000098	C00181	Xylose	90.018	248.165	118.897	135.684	194.528	191.115	324.271	439.009	314.094	457.965	541.095	411.231	314.226	406.204	390.572	467.803	575.748	408.606	320.741	319.003	363.105
Carbohydrates	HMDB0000621	C00309	Ribulose	46.976	132.478	52.98	67.689	99.013	83.513	209.21	323.798	241.421	313.871	357.089	285.693	182.528	291.554	256.139	337.742	360.953	268.359	210.242	225.293	242.754
Carbohydrates	HMDB0001644	C00310	Xylulose	36.821	104.996	44.146	51.755	77.374	65.273	169.054	272.517	194.096	255.628	293.222	233.965	155.127	243.975	220.588	275.308	295.903	215.681	169.615	181.061	189.486
Carbohydrates	HMDB0000849	C00507	Rhamnose	11.08	10.587	19.004	17.849	18.436	14.469	10.035	13.219	13.386	14.72	12.204	14.753	13.44	10.726	14.319	16.384	14.539	16.463	14.786	14.643	15.952
Carbohydrates	HMDB0000660	C02336	Fructose	3.674	8.576	2.684	3.156	4.979	4.099	19.869	25.582	14.204	20.322	19.62	18.02	14.94	23.336	17.873	21.017	23.769	20.267	14.833	14.346	15.044
Carbohydrates	HMDB0000215	C00140	N-Acetyl-D-glucosamine	77.158	54.562	75.729	45.596	58.89	65.216	76.747	80.41	45.046	59.615	86.845	53.107	60.661	74.362	73.026	59.843	80.517	49.433	79.507	82.538	86.403
Carbohydrates	HMDB0000956	C00898	Tartaric acid	0.141	0.102	0.185	0.116	0.153	0.182	0.12	0.151	0.104	0.148	0.21	0.199	0.14	0.146	0.188	0.171	0.203	0.221	0.15	0.145	0.266
Carbohydrates	HMDB0000124	C00085	Fructose 6-phosphate	655.262	1543.148	464.107	867.588	832.017	568.912	5973.182	4278.632	4047.518	4824.621	3200.548	3726.612	4648.449	4028.33	4434.15	4094.247	3642.861	4762.798	3046.131	2723.618	2632.693
Carbohydrates	HMDB0001548	C00117	Ribose 5-	1087.803	1571.567	1206.367	1545.851	1274.992	1268.599	2369.172	2072.688	2179.984	2550.75	2053.297	2057.818	2064.344	1555.348	2290.809	1982.063	1706.786	2266.558	1681.348	1732.717	1751.565
Carbohydrates	HMDB0001401	C00092	Glucose 6-	1059.233	1845.386	862.047	1198.039	1138.277	871.1	4946.837	4561.411	3746.465	5247.094	3477.856	3939.398	4440.931	3731.667	4536.227	3994.683	3425.718	4743.685	3075.453	3016.979	2891.63
Carbohydrates	HMDB0000565	C00880	Galactonic acid	2.88	1.939	2.262	2.663	2.486	2.169	1.75	2.068	1.711	2.103	2.255	2.271	2.061	1.857	2.264	1.936	2.616	2.029	2.073	2.298	2.423
Carbohydrates	HMDB0000150	C00198	Gluconolactone	21.544	18.346	19.573	25.566	17.985	22.503	19.664	27.075	15.337	23.171	24.661	22.7	16.535	19.966	21.184	16.841	30.34	23.229	22.507	20.961	26.721
Carbohydrates	HMDB0000613	NA	Erythronic acid	0.379	0.385	0.371	0.533	0.438	0.425	0.223	0.487	0.203	0.44	0.582	0.36	0.315	0.257	0.34	0.272	0.524	0.486	0.389	0.393	0.513
Carbohydrates	HMDB0000867	C01685	Ribonic acid	1.988	1.352	1.845	2.209	1.671	1.43	1.24	2.346	0.861	1.268	2.288	2.011	1.541	1.342	1.832	1.303	2.358	1.667	1.828	1.815	1.823
Carbohydrates	HMDB0001262	C01835	Maltotriose	0.537	0.914	0.293	0.348	0.696	0.713	0.811	1.126	0.0242	0.311	0.374	0.261	0.965	0.847	0.431	0.527	0.684	0.242	1.082	0.645	0.68
Carbohydrates	HMDB0001051	C02154	Glyceraldehyde	51.613	66.707	33.933	27.743	47.195	37.7	81.15	101.861	60.307	83.122	75.288	76.773	66.691	83.864	87.067	82.701	82.593	84.586	76.164	78.762	80.401
Carnitines	HMDB0000062	C00318	Carnitine	59.055	54.677	51.502	55.708	44.312	52.937	39.654	41.186	35.992	42.258	44.781	37.967	40.338	40.268	51.005	41.296	48.152	43.675	45.981	46.497	48.473
Carnitines	HMDB0000201	C02571	Acetylcarnitine	9.834	11.127	11.036	13.617	10.162	11.483	7.468	7.572	8.131	8.132	9.342	8.48	8.825	8.634	9.55	9.558	10.164	9.815	9.905	10.315	9.791
Carnitines	HMDB0002095	NA	Malonylcarnitine	0.128	0.14	0.134	0.176	0.123	0.135	0.123	0.122	0.118	0.125	0.107	0.132	0.118	0.124	0.139	0.116	0.137	0.131	0.137	0.137	0.129
Carnitines	HMDB0002013	C02862	Butyrylcarnitine	0.752	0.669	0.717	0.794	0.629	0.723	0.499	0.501	0.454	0.633	0.727	0.711	0.478	0.483	0.634	0.653	0.709	0.775	0.636	0.64	0.678
Carnitines	HMDB0000378	NA	2-Methylbutyroylcarnitine	0.526	0.513	0.479	0.577	0.48	0.506	0.71	0.697	0.569	0.697	0.729	0.681	0.732	0.658	0.726	0.665	0.728	0.691	0.637	0.64	0.664
Carnitines	HMDB0013128	NA	Valerylcarnitine	0.009	0.012	0.007	0.014	0.005	0.011	0.014	0.006	0.007	0.016	0.025	0.02	0.022	0.017	0.024	0.022	0.018	0.022	0.021	0.016	0.02
Carnitines	HMDB0000688	NA	Isovalerylcarnitine	0.326	0.351	0.274	0.353	0.264	0.255	0.358	0.266	0.249	0.291	0.281	0.257	0.333	0.275	0.249	0.235	0.312	0.249	0.29	0.269	0.284
Carnitines	NA	NA	3-Hydroxylisovalerylcarnitine	0.395	0.366	0.358	0.396	0.317	0.347	0.374	0.36	0.308	0.396	0.383	0.347	0.373	0.365	0.389	0.362	0.394	0.369	0.385	0.38	0.369
Carnitines	HMDB0013130	NA	Glutarylcarnitine	0.02	0.013	0.016	0.014	0.01	0.014	0.012	0.015	0.011	0.016	0.021	0.013	0.013	0.012	0.015	0.015	0.015	0.014	0.016	0.01	0.016
Carnitines	HMDB0000705	NA	Hexanylcarnitine	0.209	0.178	0.185	0.218	0.155	0.179	0.142	0.134	0.154	0.195	0.221	0.255	0.187	0.147	0.169	0.207	0.193	0.229	0.181	0.186	0.182
Carnitines	HMDB0061677	NA	Adipoylcarnitine	0.003	0.002	0.003	0.002	0.001	0.001	0.001	0.001	0.002	0.002	0.003	0.002	0.001	0.002	0.002	0.002	0.002	0.004	0.002	0.003	0.004
Carnitines	HMDB0000791	C02838	Octanoylcarnitine	0.02	0.011	0.017	0.024	0.017	0.019	0.005	0.005	0.006	0.008	0.011	0.014	0.006	0.005	0.007	0.014	0.008	0.011	0.012	0.009	0.013
Carnitines	HMDB0000651	NA	Decanoylcarnitine	0.048	0.047	0.045	0.051	0.042	0.049	0.042	0.042	0.042	0.044	0.048	0.041	0.043	0.039	0.039	0.042	0.04	0.04	0.042	0.042	0.043
Carnitines	HMDB0002250	NA	Dodecanoylcarnitin	0.143	0.115	0.16	0.198	0.117	0.147	0.08	0.084	0.083	0.101	0.124	0.103	0.081	0.076	0.075	0.078	0.083	0.082	0.097	0.101	0.106
Carnitines	HMDB0005066	NA	Tetradecanoylcarnitine	2.097	1.435	1.934	2.296	1.403	2.005	0.539	0.55	0.499	0.68	0.843	0.758	0.496	0.433	0.473	0.493	0.542	0.576	0.937	0.988	0.979
Carnitines	HMDB0000222	C02990	Palmitoylcarnitine	6.723	5.032	5.85	6.547	4.967	6.713	1.716	1.521	1.42	2.071	2.113	2.637	2.015	1.466	1.674	1.614	1.943	2.45	3.032	3.203	3.164
Carnitines	HMDB0005065	NA	Oleylcarnitine	1.5	1.062	1.091	1.327	0.916	1.38	0.253	0.229	0.196	0.316	0.296	0.333	0.238	0.253	0.249	0.295	0.302	0.345	0.537	0.566	0.556
Carnitines	HMDB0000848	NA	Stearylcarnitine	0.778	0.63	0.586	0.656	0.663	0.825	0.22	0.148	0.17	0.306	0.186	0.405	0.305	0.235	0.317	0.26	0.31	0.32	0.474	0.432	0.335
Carnitines	HMDB0013133	NA	Methylmalonylcarnitine	0.589	0.695	0.603	0.65	0.44	0.684	0.347	0.546	0.237	0.515	0.453	0.471	0.528	0.453	0.523	0.501	0.545	0.526	0.592	0.696	0.47
Carnitines	HMDB0000824	C03017	Propionylcarnitine	1.118	0.931	0.989	1.135	0.921	1.002	1.632	1.612	1.675	1.8	1.752	1.568	1.869	1.561	1.72	1.937	1.656	1.521	1.454	1.466	1.514
Carnitines	HMDB0006469	NA	Linoleylcarnitine	0.037	0.019	0.026	0.026	0.019	0.03	0.004	0.005	0.006	0.008	0.005	0.006	0.005	0.004	0.005	0.006	0.005	0.007	0.01	0.01	0.014
Eicosanoids	HMDB0003876	C04742	15-HETE	1.04	1.045	1.273	0.104	0.104	0.104	19.514	19.818	24.268	16.317	31.393	18.696	24.69	25.697	28.148	28.236	24.189	20.54	19.406	18.542	16.363
Fatty Acids	HMDB0010720	C01771	2-Butenoic acid	2.71	3.897	0.2148	0.2148	3.939	2.764	2.901	5.403	2.148	3.85	4.069	2.243	0.2148	4.813	3.701	2.928	4.639	4.237	5.117	4.363	3.701
Fatty Acids	HMDB0031158	NA	2-Methy-4-pentenoic acid	4.668	5.352	4.121	4.236	4.429	5.385	3.975	3.597	4.061	3.411	4.561	3.663	3.871	3.17	4.366	4.302	4.647	3.901	4.533	4.806	3.714
Fatty Acids	HMDB0000784	C08261	Azelaic acid	0.499	0.511	0.429	0.722	0.585	0.504	0.658	0.525	0.75	0.807	0.634	0.511	0.572	0.559	0.503	0.549	0.531	0.675	0.588	0.555	0.557
Fatty Acids	HMDB0000792	C08277	Sebacic acid	0.337	0.32	0.315	0.35	0.298	0.334	0.391	0.344	0.389	0.393	0.37	0.34	0.366	0.325	0.314	0.321	0.31	0.327	0.333	0.35	0.351
Fatty Acids	HMDB0000634	C02226	Citraconic acid	1.404	1.343	1.296	1.662	1.442	1.372	23.965	30.129	21.771	26.736	34.149	27.578	23.704	25.069	20.767	25.408	30.569	26.348	16.881	17.913	20.454
Fatty Acids	HMDB0001844	NA	Methylsuccinic	0.868	0.718	0.779	0.859	0.739	0.72	1.188	1.116	0.96	1.186	1.314	1.118	1.029	1.099	1.121	1.033	1.265	1.145	1.042	0.951	1.026
Fatty Acids	HMDB0000448	C06104	Adipic acid	1.171	1.237	1.382	1.464	1.155	1.271	1.52	1.337	1.779	1.396	1.227	1.275	1.071	0.783	0.703	1.114	1.114	1.443	0.921	1.087	1.346

Fatty Acids	HMDB0000752	NA	Methylglutaric acid	0.072	0.04	0.055	0.073	0.056	0.076	0.079	0.087	0.086	0.102	0.102	0.059	0.06	0.076	0.06	0.069	0.083	0.093	0.066	0.058	0.091
Fatty Acids	HMDB0000555	NA	3-Methyladipic	0.624	0.612	0.78	0.649	0.653	0.68	0.681	0.783	0.601	0.579	0.705	0.667	0.589	0.571	0.566	0.579	0.742	0.644	0.646	0.695	0.733
Fatty Acids	HMDB0000893	C08278	Suberic acid	0.461	0.348	0.331	0.505	0.498	0.424	0.61	0.441	0.588	0.493	0.488	0.338	0.474	0.41	0.423	0.402	0.432	0.622	0.407	0.412	0.481
Fatty Acids	NA	NA	4-Methylhexanoic acid	0.381	0.36	0.394	0.032	0.32	0.35	0.355	0.338	0.352	0.357	0.356	0.339	0.406	0.37	0.347	0.381	0.032	0.36	0.335	0.321	0.376
Fatty Acids	HMDB0000666	C17714	Heptanoic acid	0.226	0.339	0.233	0.246	0.166	0.212	0.194	0.302	0.209	0.272	0.294	0.162	0.186	0.129	0.248	0.204	0.252	0.288	0.246	0.398	0.312
Fatty Acids	HMDB0000482	C06423	Octanoic acid	17.473	18.9	21.052	17.601	17.274	18.305	15.644	15.323	17.183	17.224	19.611	17.905	14.034	14.316	13.293	17.616	18.217	14.785	17.078	18.401	19.429
Fatty Acids	HMDB0000847	C01601	Nonanoic acid	3.019	4.071	3.573	3.17	3.013	3.332	3.189	3.031	3.636	3.768	4.267	3.454	2.841	3.107	2.61	3.366	3.555	3.572	3.895	3.784	4.157
Fatty Acids	HMDB0000511	C01571	Decanoic acid	3.095	4.002	3.457	3.263	2.904	3.327	2.889	3.252	2.938	3.293	3.654	3.371	2.473	2.162	2.272	3.637	3.114	2.246	2.61	3.438	3.238
Fatty Acids	HMDB0033724	C13910	Undecylenic acid	0.031	0.09	0.067	0.033	0.043	0.058	0.042	0.043	0.064	0.051	0.086	0.021	0.042	0.028	0.074	0.064	0.044	0.05	0.04	0.054	0.039
Fatty Acids	HMDB0000947	C17715	Undecanoic acid	0.396	0.536	0.533	0.503	0.444	0.399	0.437	0.52	0.501	0.513	0.484	0.502	0.449	0.457	0.412	0.51	0.462	0.431	0.47	0.609	0.465
Fatty Acids	HMDB0000638	C02679	Dodecanoic acid	9.018	8.906	9.398	11.529	7.944	8.235	11.663	9.912	10.452	10.094	7.403	10.295	7.28	8.128	10.652	8.73	9.542	7.009	9	9.857	8.113
Fatty Acids	HMDB0000910	C17076	Tridecanoic acid	1.325	1.25	1.462	1.538	1.188	1.321	1.365	1.29	1.138	1.329	1.247	1.284	1.016	0.955	1.148	1.037	1.238	1.162	1.18	1.291	1.254
Fatty Acids	HMDB0002000	C08322	Myristoleic acid	2.765	2.648	3.056	3.475	2.529	3.058	2.31	2.351	2.24	2.474	2.618	2.352	1.792	1.893	2.204	2.197	2.306	2.074	2.445	2.629	2.428
Fatty Acids	HMDB0062248	NA	9E-tetradecenoic acid	1.048	1.099	1.291	1.537	1.131	1.049	0.712	0.603	0.642	0.9	0.818	0.739	0.557	0.492	0.66	0.685	0.722	0.697	0.929	0.892	0.846
Fatty Acids	HMDB0000806	C06424	Myristic acid	86.832	69.693	86.282	71.134	72.536	94.139	73.445	69.804	81.959	79.284	74.806	81.903	50.928	67.335	75.04	77.727	75.015	58.198	78.877	79.19	85.838
Fatty Acids	HMDB0000826	C16537	Pentadecanoic acid	7.62	6.305	7.924	4.604	9.403	7.324	7.336	7.428	9.935	6.924	6.873	8.282	5.079	7.953	8.257	9.243	8.99	6.132	9.517	7.881	8.142
Fatty Acids	HMDB0003229	C08362	Palmitoleic acid	75.621	68.034	74.667	76.891	68.866	97.211	84.902	79.98	83.218	83.948	72.698	88.514	54.785	81.495	76.89	74.16	84.669	63.699	82.204	82.883	81.667
Fatty Acids	HMDB0060038	NA	10Z-Heptadecenoic acid	2.994	4.268	4.658	4.66	3.896	7.06	5.702	4.586	5.411	4.521	3.332	4.644	3.767	5.143	5.086	5.492	5.143	4.099	4.712	4.839	4.582
Fatty Acids	HMDB0001388	C06427	alpha-Linolenic	0.966	1.042	1.07	0.701	0.937	1.379	1.369	1.179	1.042	1.073	1.004	1.083	0.984	1.089	0.927	0.735	0.758	0.716	1.199	1.148	1.053
Fatty Acids	HMDB0000673	C01595	Linoleic acid	66.699	94.783	94.717	90.804	82.175	82.034	152.978	75.739	131.015	128.579	67.645	104.497	117.992	142.141	103.07	117.03	132.118	77.602	111.126	117.509	72.036
Fatty Acids	NA	NA	10,13-Nonadecadienoic acid	2.527	4.387	2.783	1.451	0.092	1.218	9.502	3.627	2.984	5.709	0.27	6.727	5.862	4.629	1.395	4.002	7.653	3.852	1.765	2.75	3.537
Fatty Acids	HMDB0001999	C06428	EPA	0.496	0.369	0.434	0.497	0.587	0.416	1.864	1.581	1.795	1.488	1.356	1.812	1.086	1.568	1.432	1.576	1.497	1.2	1.188	1.282	1.248
Fatty Acids	HMDB0001043	C00219	Arachidonic acid	2.769	3.495	2.611	1.856	3.362	3.856	8.91	6.795	9.316	9.276	6.8	8.917	8.752	10.061	8.019	7.947	10.597	8.222	7.889	6.664	5.994
Fatty Acids	HMDB0002925	C03242	Dihomo-gamma-linolenic acid	0.581	0.828	0.432	0.393	0.735	0.733	1.879	1.037	2.197	1.724	0.898	1.828	1.412	2.179	1.73	1.832	2.117	1.44	1.153	1.221	1.078
Fatty Acids	HMDB0002183	C06429	DHA	13.576	17.975	11.57	11.314	16.961	14.578	36.6	24.755	28.092	32.145	22.357	28.197	31.409	30.549	28.379	27.909	34.019	25.869	27.476	28.117	21.427
Fatty Acids	HMDB0006528	C16513	DPA	9.768	14.952	8.676	8.726	13.476	11.887	28.751	17.264	25.22	27.483	17.491	27.817	27.287	25.843	25.711	25.438	30.666	19.091	22.188	20.065	16.327
Fatty Acids	HMDB0001976	NA	DPAn-6	0.436	0.491	0.332	0.466	0.517	0.528	0.723	0.423	0.589	0.778	0.392	0.662	0.795	0.517	0.614	0.703	0.836	0.509	0.536	0.539	0.371
Fatty Acids	HMDB0002226	C16527	Adrenic acid	0.814	1.705	1.111	0.48	0.47	0.356	4.971	1.198	4.214	3.645	1.974	3.944	3.308	3.599	3.325	2.39	5.267	1.792	2.601	2.652	2.073
Fatty Acids	HMDB0000426	C00815	Citramalic acid	0.189	0.197	0.159	0.214	3.157	0.171	0.287	0.245	0.375	0.278	0.261	0.248	0.187	0.208	0.224	0.255	0.227	0.226	0.25	0.227	0.239
Fatty Acids	HMDB0000207	C00712	Oleic acid	518.133	507.558	581.527	400.283	518.161	617.812	635.751	391.38	666.974	562.334	345.127	594.286	481.947	693.893	599.909	573.115	632.407	384.502	631.055	542.706	447.671
Fatty Acids	HMDB0002074	NA	2,2-Dimethylsuccinic	0.232	0.23	0.205	0.235	0.221	0.206	0.252	0.274	0.235	0.255	0.274	0.245	0.211	0.281	0.221	0.245	0.25	0.206	0.228	0.232	0.243
Fatty Acids	HMDB0000529	NA	5Z-Dodecenoic	0.303	0.294	0.387	0.378	0.275	0.319	0.234	0.248	0.213	0.273	0.255	0.233	0.178	0.186	0.256	0.236	0.227	0.221	0.249	0.298	0.224
Fatty Acids	HMDB0034297	C08365	Ricinoleic acid	0.114	0.116	0.104	0.127	0.103	0.107	0.178	0.212	0.188	0.172	0.221	0.194	0.196	0.219	0.222	0.19	0.19	0.201	0.159	0.162	0.174
Fatty Acids	HMDB0061706	NA	12-Hydroxystearic acid	0.25	0.199	0.259	0.279	0.269	0.253	0.183	0.18	0.18	0.303	0.219	0.225	0.201	0.2	0.193	0.317	0.187	0.258	0.267	0.239	0.263
Fatty Acids	HMDB0012328	NA	Palmitelaidic acid	2.812	1.788	1.957	1.762	3.015	2.703	3.577	2.393	3.789	2.879	2.154	3.628	2.147	3.028	2.727	3.335	4.031	1.872	3.498	2.825	1.986
Fatty Acids	HMDB0006270	NA	Linoelaidic acid	4.877	2.078	2.869	3.279	3.298	5.122	4.412	3.197	3.299	4.644	1.634	4.013	3.07	4.349	3.68	4.169	4.099	2.155	3.816	3.271	2.77
Fatty Acids	NA	NA	2,2-Dimethyladipic acid	0.043	0.046	0.051	0.051	0.05	0.045	0.044	0.063	0.037	0.058	0.054	0.061	0.044	0.045	0.051	0.048	0.065	0.042	0.053	0.055	0.059
Imidazoles	HMDB0002271	C20522	Imidazolepropionic acid	0.239	0.26	0.217	0.281	0.151	0.204	0.161	0.123	0.118	0.155	0.168	0.158	0.131	0.133	0.147	0.107	0.14	0.15	0.185	0.166	0.172
Indoles	HMDB0000671	C02043	Indolelactic acid	0.192	0.216	0.191	0.221	0.248	0.14	0.138	0.127	0.106	0.152	0.266	0.137	0.051	0.142	0.072	0.101	0.136	0.156	0.232	0.096	0.232
Indoles	HMDB0060484	C00331	Indole-3-pyruvic	0.201	0.168	0.144	0.157	0.2	0.2	0.212	0.174	0.21	0.267	0.199	0.183	0.153	0.175	0.169	0.221	0.187	0.151	0.191	0.201	0.158
Nucleotides	HMDB0000045	C00020	AMP	2030.222	1791.221	1758.611	1699.093	1659.333	1781.369	1047.315	1232.813	1351.954	1454.204	1292.379	1487.96	1397.51	1144.644	1254.807	1381.97	1182.505	1294.476	1518.069	1457.538	1432.251
Nucleotides	HMDB0001397	C00144	GMP	4229.876	3472.678	3116.493	4010.798	3057.885	3307.276	1278.461	2826.458	2162.938	2753.35	3212.835	3627.33	2558.961	1672.748	2700.576	2490.744	2062.885	3770.625	2701.507	2802.085	3044.455
Nucleotides	HMDB0000939	C00021	SAH	39.169	28.295	26.737	30.493	27.566	29.308	11.383	21.5	15.81	20.317	18.557	18.474	17.602	14.182	14.95	17.872	13.918	17.115	20.425	19.199	21.724
Organic Acids	HMDB0000700	C01013	Hydroxypropionic acid	5.337	4.554	4.905	5.429	5.274	6.535	8.786	9.238	9.596	8.638	10.043	6.358	6.544	7.721	5.521	9.184	4.587	8.477	4.37	7.542	6.897
Organic Acids	HMDB0000190	C00186	Lactic acid	6741.953	5763.656	6730.03	6365.127	4746.026	5746.297	4294.776	3895.04	2985.403	4037.965	4945.203	4827.32	4788.279	4144.261	6038.717	4440.571	4714.071	4258.73	5235.015	4630.938	6916.639
Organic Acids	HMDB0000156	C00149	Malic acid	533.769	467.271	487.171	536.69	394.927	448.136	478.644	495.994	409.703	479.585	530.105	487.761	402.651	452.918	482.308	480.858	553.214	469.237	469.725	477.828	469.15
Organic Acids	HMDB0001870	C00180	Benzoic acid	0.418	0.391	0.181	0.33	0.414	0.511	0.64	0.252	0.605	0.67	0.888	0.387	2.013	0.607	0.236	0.414	0.275	0.377	0.459	0.796	0.504
Organic Acids	HMDB0000134	C00122	Fumaric acid	206.266	194.18	180.191	185.191	145.706	180.919	180.952	172.185	145.808	159.094	175.891	173.89	164.033	194.495	179.668	180.05	205.181	177.604	170.11	182.343	163.278
Organic Acids	HMDB0000661	C00489	Glutaric acid	1.488	1.279	1.193	1.423	1.084	1.335	1.741	1.654	1.428	1.662	1.81	1.909	1.446	1.657	1.723	1.654	1.789	1.628	1.471	1.549	1.446
Organic Acids	HMDB0000072	C02341	Aconitic acid	51.788	58.171	61.592	71.335	56.606	57.645	138.303	116.898	108.942	148.226	163.331	168.855	159.486	140.804	127.043	145.623	164.051	152.768	115.595	119.026	105.424
Organic Acids	HMDB0000019	C00141	alpha-Ketoisovaleric acid	2.158	2.222	4.126	2.737	2.507	3.675	3.45	0.0925	4.14	1.142	2.792	3.713	1.814	2.811	2.999	0.933	0.0925	0.925	3.498	5.071	2.036
Organic Acids	HMDB0000695	C00233	Ketoleucine	2.254	1.896	2.195	2.501	1.772	2.105	1.719	1.676	1.752	1.497	2.187	1.755	0.977	1.433	1.627	1.243	1.266	1.239	2.019	2.022	1.988
Organic Acids	HMDB0000491	C00671	3-Methyl-2-oxopentanoic acid	1.324	1.706	1.797	1.814	1.144	2.008	1.581	1.341	1.681	1.273	1.678	1.065	1.032	1.544	0.762	1.492	0.909	1.47	1.21	1.849	1.532
Organic Acids	HMDB0000202	C02170	Methylmalonic	67.971	65.663	72.722	76.047	57.228	64.885	46.662	47.77	40.68	57.181	61.676	51.952	56.733	41.04	60.102	50.808	56.663	51.35	56.98	57.288	54.22
Organic Acids	HMDB0000606	C02630	2-Hydroxyglutaric acid	71.358	61.88	61.477	67.955	53.528	61.009	57.182	62.519	47.991	60.936	66.485	62.19	58.266	62.349	63.641	61.07	70.303	57.17	63.661	62.122	61.705

Organic Acids	HMDB0000128	C00581	Guanidoacetic acid	2.047	2.025	1.426	1.38	1.358	1.845	1.209	1.771	0.901	1.143	0.986	0.852	1.36	1.394	1.769	0.968	2.347	1.057	1.196	0.957	1.555
Organic Acids	HMDB0003070	C00493	Shikimic acid	4.241	4.291	3.199	3.426	3.311	2.732	4.163	3.742	2.91	4.327	4.114	3.087	4.464	3.956	4.28	4.172	3.526	3.253	4.443	4.579	4.2
Organic Acids	HMDB0000357	C01089	3-Hydroxybutyric acid	6.205	5.746	5.973	7.468	4.669	5.845	4.978	4.842	4.943	5.84	6.851	5.402	4.693	5.429	6.06	5.219	6	5.953	5.662	5.415	5.231
Organic Acids	HMDB0000008	C05984	2-Hydroxybutyric acid	0.333	0.263	0.086	0.28	0.161	0.111	0.271	0.229	0.116	0.261	0.243	0.269	0.315	0.226	0.314	0.32	0.203	0.265	0.213	0.282	0.33
Organic Acids	HMDB0000620	C02214	Glutaconic acid	137.397	154.994	129.224	193.12	135.527	166.396	105.531	136.495	103.002	160.34	205.755	163.916	112.33	113.523	138.429	162.837	151.682	158.525	121.913	132.919	141.586
Organic Acids	HMDB0000691	C00383	Malonic acid	0.191	0.196	0.206	0.279	0.219	0.194	0.295	0.248	0.216	0.313	0.357	0.237	0.248	0.196	0.213	0.226	0.216	0.256	0.288	0.278	0.376
Organic Acids	HMDB0002329	C00209	Oxalic acid	6.7	7.179	6.916	6.934	8.211	8.788	9.23	11.569	13.191	9.322	14.403	8.777	8.029	9.252	7.645	8.989	10.044	8.112	10.806	10.958	13.147
Organic Acids	HMDB0000176	C01384	Maleic acid	9.894	9.225	10.941	9.744	9.292	10.239	10.796	9.812	7.71	8.154	14.059	7.651	8.189	9.557	7.34	8.26	14.206	11.674	10.453	9.8	11.659
Organic Acids	HMDB0000060	C00164	Acetoacetic acid	16.035	14	13.722	14.334	13.081	15.696	25.484	30.607	18.317	23.04	23.761	28.064	19.341	21.319	27.763	25.173	20.844	25.876	24.65	21.4	21.295
Organic Acids	HMDB0000094	C00158	Citric acid	54.026	61.486	59.472	80.943	61.477	57.156	153.793	123.122	111.077	145.182	166.106	187.373	183.821	145.399	161.346	162.483	169.83	170.246	116.348	122.959	122.584
Organic Acids	HMDB0000193	C00311	Isocitric acid	0.514	0.359	0.618	0.206	0.288	0.589	1.068	1.509	0.823	2.505	1.205	0.633	2.415	0.865	1.109	0.446	1.663	0.966	1.547	1.211	1.351
Organic Acids	HMDB0000243	C00022	Pyruvic acid	440.635	472.627	471.988	557.939	391.501	461.555	392.518	386.024	338.878	394.147	356.539	364.188	353.583	318.658	363.298	351.996	360.363	334.431	376.933	371.11	398.727
Organic Acids	HMDB0000208	C00026	Oxoglutaric acid	3.262	3.195	2.076	1.574	2.319	1.632	3.8	3.111	3.461	3.049	3.368	4.328	3.801	4.062	4.223	4.097	4.252	4.178	3.538	3.445	3.439
Organic Acids	HMDB0000254	C00042	Succinic acid	152.008	148.031	154.832	185.255	120.575	148.656	100.212	100.772	91.215	122.897	128.365	116.89	115.292	96.507	141.974	103.75	119.97	109.413	133.77	124.97	119.191
Organic Acids	HMDB0000729	NA	alpha-Hydroxyisobutyric acid	0.011	0.001	0.01	0.037	0.047	0.023	0.035	0.018	0.035	0.031	0.004	0.036	1.00E-04	0.028	0.032	0.041	0.015	0.012	0.012	0.015	0.022
Peptides	HMDB0000194	C01262	Anserine	1.055	1.44	0.831	0.571	1.09	0.677	0.643	0.837	0.942	1.14	0.974	0.643	0.925	1.257	1.057	0.726	0.667	1.042	0.613	0.974	0.861
Peptides	HMDB0000033	C00386	Carnosine	29.833	20.851	21.392	20.825	21.762	26.385	20.975	19.281	12.807	23.732	23.107	19.837	21.773	17.824	20.301	26.175	27.023	22.064	19.716	22.964	24.772
Peptides	HMDB0000721	NA	Glycylproline	2.406	1.45	1.621	0.95	1.224	1.598	1.346	1.393	0.848	2.032	1.732	1.332	1.653	1.827	1.712	1.549	2.45	1.742	1.591	1.908	1.841
Peptides	HMDB0000759	C02155	Glycyleucine	7.739	4.876	4.015	5.075	2.821	5.133	4.002	2.575	3.553	3.171	3.515	3.138	3.691	2.087	3.374	2.81	3.274	5.241	3.391	4.833	4.801
Peptides	HMDB0006248	C03740	gamma-Glutamylalanine	25.774	27.343	21.769	25.108	23.952	23.627	19.849	25.388	24.665	22.552	23.283	23.74	26.354	26.197	28.709	30.394	36.552	27.541	25.222	25.127	25.385
Phenols	HMDB0000822	C11527	p-Hydroxymandelic	0.91	0.843	0.0724	0.813	0.724	0.76	0.776	0.0724	0.893	0.0724	0.971	0.735	0.765	0.811	0.786	0.741	0.736	0.881	0.788	1.021	0.775
Phenols	HMDB0000118	C05582	Homovanillic acid	2.386	2.034	1.703	2.063	1.664	1.509	2.432	2.256	1.645	2.421	2.106	2.12	2.294	1.925	1.904	1.617	1.71	2.293	1.871	2.119	2.083
Phenylpropanoic Acids	HMDB0000779	NA	Phenyllactic acid	4.03	2.419	3.092	3.753	2.18	3.03	2.15	2.371	2.43	2.64	2.578	2.6	2.096	1.629	1.524	1.866	1.403	2.148	1.831	2.459	2.539
Pyridines	HMDB0001488	C00253	Nicotinic acid	0.0432	0.0432	0.0432	0.0432	0.0432	0.0432	3.947	3.449	0.432	1.935	3.277	0.998	1.986	2.748	1.766	2.115	1.924	2.448	1.338	0.949	1.346
Pyridines	HMDB0002243	C10164	Picolinic acid	1.381	1.6	1.426	1.844	2.163	1.901	1.653	2.698	2.026	2.392	3.822	1.936	1.654	1.931	1.819	2.094	2.618	2.41	1.724	1.74	2.35
Pyridines	HMDB0003152	NA	N-Methylnicotinamid	1	0.166	0.564	2.09	1.038	0.308	1.219	0.817	0.542	1.361	1.171	1.387	0.839	0.67	0.752	1.826	0.821	1.382	0.467	0.959	1.111
SCFAs	HMDB0002176	C18319	Ethylmethylacetic acid	2.745	2.69	2.49	2.678	2.49	2.537	2.793	2.994	2.771	3.012	2.987	2.724	2.831	2.668	2.829	2.707	2.623	2.576	2.755	2.714	2.658
SCFAs	HMDB0000042	C00033	Acetic acid	84.073	73.978	74.809	116.233	93.573	100.974	44.187	75.433	29.289	70.804	79.018	47.486	46.695	56.307	78.007	59.698	71.347	70.086	76.222	77.534	85.677
SCFAs	HMDB0000754	NA	3-Hydroxyisovaleric	0.728	0.653	0.47	0.514	0.345	0.352	1.307	1.008	1.079	0.841	0.933	0.883	1.146	0.989	0.908	0.839	0.79	0.609	0.769	0.807	0.756
SCFAs	HMDB0000237	C00163	Propionic acid	8.283	9.06	8.472	11.784	10.394	10.032	8.732	11.32	10.461	12.019	14.597	10.299	9.876	9.326	13.369	11.215	11.433	9.979	9.901	9.506	9.464
SCFAs	HMDB0000039	C00246	Butyric acid	7.056	6.354	6.557	7.139	6.782	6.602	5.277	5.586	3.963	5.288	6.014	4.755	5.055	4.867	5.193	4.759	4.789	5.173	6.377	6.073	6.541
SCFAs	HMDB0001873	C02632	Isobutyric acid	18.417	12.424	13.02	11.586	17.375	12.742	32.491	25.582	16.425	19.748	28.122	17.463	21.71	26.332	18.93	14.436	17.438	19.812	17.958	18.487	20.276
SCFAs	HMDB0000535	C01585	Caproic acid	1.996	2.304	1.856	2.359	2.168	2.137	0.898	1.132	0.824	1.579	1.193	1.537	0.828	0.883	1.683	1.507	1.316	1.748	2.294	1.456	2.119
SCFAs	HMDB0031580	NA	2-Methylpentanoic acid	0.397	0.39	0.383	0.422	0.381	0.359	0.398	0.375	0.384	0.396	0.392	0.363	0.4	0.359	0.372	0.381	0.384	0.369	0.396	0.389	0.384
