# Supplementary material for: Metabolomic landscape of macrophage discloses an anabolic signature of dengue virus infection and antibody-dependent enhancement of viral infection
Source: PLoS Negl Trop Dis. 2024 Feb 2;18(2):e0011923. doi: 10.1371/journal.pntd.0011923 (PMC10866464; doi:10.1371/journal.pntd.0011923)
Supplement: S2 Table — (PDF) [file pntd.0011923.s010.pdf]

S2 Table : Overall differentially expressed metabolites

Class	Metabolite	Uni_P	Uni_FDR
Amino Acids	Lysine	0.020003614	0.048472335
Amino Acids	Histidine	0.03210972	0.070659635
Amino Acids	Arginine	0.00223136	0.009014694
Amino Acids	Glutamine	0.675830573	0.798349566
Amino Acids	Glutamic acid	0.410593777	0.556643913
Amino Acids	beta-Alanine	0.608621668	0.727162987
Amino Acids	Alanine	0.012327106	0.031924044
Amino Acids	GABA	0.006369248	0.017869279
Amino Acids	Serine	0.030966223	0.069501968
Amino Acids	Threonine	0.063846705	0.125573689
Amino Acids	Creatine	1.27935E-05	0.000152017
Amino Acids	Aminocaproic acid	0.870306657	0.925273393
Amino Acids	Methylcysteine	0.043438472	0.090459498
Amino Acids	Tyrosine	0.007949557	0.021410808
Amino Acids	Asparagine	0.056199752	0.112399504
Amino Acids	Phenylalanine	0.000201474	0.001453493
Amino Acids	Kynurenine	0.013832951	0.034928202
Amino Acids	Aspartic acid	0.495580554	0.617946123
Amino Acids	Aminoadipic acid	0.835942097	0.902996276
Amino Acids	N-Acetyalanine	0.143577175	0.245784656
Amino Acids	N-Acetyaspartic acid	0.000161874	0.001307942
Amino Acids	N-Phenylacetylphenylalanine	0.384162027	0.531511845
Amino Acids	Glycine	0.151178372	0.25662211
Amino Acids	Citrulline	0.895798407	0.942454574
Amino Acids	alpha-Aminobutyric acid	0.366720389	0.514427213
Amino Acids	Proline	0.136766277	0.238161965
Amino Acids	Acetylglycine	0.08117814	0.154697965
Amino Acids	Pipecolic acid	0.697596256	0.809853124
Amino Acids	N-Acetylserine	0.303719466	0.451112737
Amino Acids	N-Acetylglutamine	0.322543033	0.462082926
Amino Acids	Valine	0.013123729	0.033556876
Amino Acids	Pyroglutamic acid	0.282535891	0.429114662
Amino Acids	5-Aminolevulinic acid	0.419008022	0.560527287
Amino Acids	Methionine	0.546920364	0.661544392
Amino Acids	Isoleucine	0.020156813	0.048472335
Amino Acids	Leucine	0.002918846	0.009826782
Amino Acids	Tryptophan	0.001598649	0.007509932
Amino Acids	Phenylacetylglutamine	0.45970595	0.604773562
Amino Acids	N-Acetylhistidine	0.972641694	0.977480708
Amino Acids	1-Methylhistidine	0.311785535	0.456381726
Amino Acids	4-Hydroxyproline	0.005959272	0.016954547
Amino Acids	Ornithine	0.460300839	0.604773562
Amino Acids	Cystine	0.296302535	0.443356386
Amino Acids	Sarcosine	0.00034456	0.002245198
Amino Acids	Dimethylglycine	0.776129106	0.875360649
Amino Acids	Homoserine	0.231692197	0.362804835
Amino Acids	Homocitrulline	0.197316242	0.322421072
Amino Acids	N-Acetyl-L-methionine	0.478629447	0.608070115
Benzenoids	Phenylpyruvic acid	0.000894856	0.004885432
Benzenoids	Mandelic acid	0.695010711	0.809853124
Benzoic Acids	m-Aminobenzoic acid	0.468215954	0.604773562
Benzoic Acids	Hippuric acid	0.001120718	0.005957501
Benzoic Acids	4-Aminohippuric acid	2.45484E-06	4.95878E-05

Benzoic Acids	Phthalic acid	0.1191708	0.214933051
Bile Acids	TCA	0.492116315	0.617437861
Bile Acids	TDCA	0.828439627	0.900547661
Bile Acids	TCDCA	0.00023608	0.001589606
Bile Acids	GHDCA	0.807841728	0.890027799
Bile Acids	CA	0.319225718	0.462082926
Bile Acids	CDCA	0.032531416	0.070659635
Bile Acids	GCDCA	0.465046019	0.604773562
Bile Acids	GDCA	0.25498635	0.390206384
Bile Acids	6,7-DiketoLCA	0.35434219	0.500539318
Carbohydrates	Glucaric acid	0.919825006	0.957755934
Carbohydrates	Glyceric acid	0.00067327	0.003777795
Carbohydrates	N-Acetylneuraminic acid	0.254395809	0.390206384
Carbohydrates	Melibiose	2.97613E-08	1.50295E-06
Carbohydrates	Glucose	0.0025523	0.009523424
Carbohydrates	Xylose	3.81536E-05	0.000385352
Carbohydrates	Ribulose	4.41896E-06	6.86638E-05
Carbohydrates	Xylulose	3.10097E-06	5.69451E-05
Carbohydrates	Rhamnose	0.393499401	0.537302946
Carbohydrates	Fructose	2.92318E-07	6.56092E-06
Carbohydrates	N-Acetyl-D-glucosamine	0.858007328	0.921901491
Carbohydrates	Tartaric acid	0.322377873	0.462082926
Carbohydrates	Fructose 6-phosphate	8.97328E-08	2.58943E-06
Carbohydrates	Ribose 5-phosphate	2.83376E-05	0.000301273
Carbohydrates	Glucose 6-phosphate	4.72156E-08	1.70533E-06
Carbohydrates	Galactonic acid	0.103492245	0.190049396
Carbohydrates	Gluconolactone	0.882634496	0.933466849
Carbohydrates	Erythronic acid	0.686632	0.806393395
Carbohydrates	Ribonic acid	0.945899829	0.969907439
Carbohydrates	Maltotriose	0.75373291	0.855359819
Carbohydrates	Glyceraldehyde	0.005088858	0.014684991
Carnitines	Carnitine	0.000374351	0.002322223
Carnitines	Acetylcarnitine	0.000186729	0.001397008
Carnitines	Malonylcarnitine	0.079419936	0.152788829
Carnitines	Butyrylcarnitine	0.123082968	0.218507233
Carnitines	2-Methylbutyroylcarnitine	0.004449583	0.013217879
Carnitines	Valerylcarnitine	0.004888715	0.01431189
Carnitines	Isovalerylcarnitine	0.488836678	0.617156306
Carnitines	3-Hydroxylisovalerylcarnitine	0.61196885	0.727162987
Carnitines	Glutarylcarnitine	0.919780635	0.957755934
Carnitines	Hexanylcarnitine	0.965534572	0.977480708
Carnitines	Adipoylcarnitine	0.865677861	0.925221841
Carnitines	Octanoylcarnitine	0.000536516	0.003096466
Carnitines	Decanoylcarnitine	0.008382372	0.021990118
Carnitines	Dodecanoylcarnitine	0.001530998	0.007363372
Carnitines	Tetradecanoylcarnitine	0.001265213	0.006553156
Carnitines	Palmitoylcarnitine	2.12113E-09	4.28468E-07
Carnitines	Oleylcarnitine	0.003335095	0.010505201
Carnitines	Stearylcarnitine	1.03463E-07	2.61244E-06
Carnitines	Methylmalonylcarnitine	0.030309375	0.068792065
Carnitines	Propionylcarnitine	5.06534E-08	1.70533E-06
Carnitines	Linoleylcarnitine	0.002781594	0.009523424
Eicosanoids	15-HETE	0.001845525	0.007942032
Fatty Acids	2-Butenoic acid	0.371918632	0.518121128
Fatty Acids	2-Methy-4-pentenoic acid	0.028413553	0.06522202
Fatty Acids	Azelaic acid	0.168500032	0.281297575

Fatty Acids	Sebacic acid	0.002601806	0.009523424
Fatty Acids	Citraconic acid	0.002753901	0.009523424
Fatty Acids	Methylsuccinic acid	7.27491E-06	0.000104967
Fatty Acids	Adipic acid	0.017372985	0.042796864
Fatty Acids	Methylglutaric acid	0.038619778	0.082117844
Fatty Acids	3-Methyladipic acid	0.156569895	0.263559323
Fatty Acids	Suberic acid	0.528650947	0.647196917
Fatty Acids	4-Methylhexanoic acid	0.539340297	0.656305663
Fatty Acids	Heptanoic acid	0.784357809	0.875360649
Fatty Acids	Octanoic acid	0.022573601	0.0536455
Fatty Acids	Nonanoic acid	0.307516823	0.453418966
Fatty Acids	Decanoic acid	0.033136676	0.071208601
Fatty Acids	Undecylenic acid	0.958460589	0.977480708
Fatty Acids	Undecanoic acid	0.340499819	0.484372982
Fatty Acids	Dodecanoic acid	0.226404379	0.35729441
Fatty Acids	Tridecanoic acid	0.002763809	0.009523424
Fatty Acids	Myristoleic acid	8.89701E-05	0.000855807
Fatty Acids	9E-tetradecenoic acid	8.77925E-06	0.000118227
Fatty Acids	Myristic acid	0.069283052	0.134569006
Fatty Acids	Pentadecanoic acid	0.780266786	0.875360649
Fatty Acids	Palmitoleic acid	0.197921846	0.322421072
Fatty Acids	10Z-Heptadecenoic acid	0.942438082	0.969907439
Fatty Acids	alpha-Linolenic acid	0.064030148	0.125573689
Fatty Acids	Linoleic acid	0.106398866	0.193626766
Fatty Acids	10,13-Nonadecadienoic acid	0.123315963	0.218507233
Fatty Acids	EPA	1.2032E-08	1.21523E-06
Fatty Acids	Arachidonic acid	0.003320625	0.010505201
Fatty Acids	Dihomo-gamma-linolenic acid	0.000113394	0.000995891
Fatty Acids	DHA	4.15993E-06	6.86638E-05
Fatty Acids	DPA	2.69891E-05	0.000301273
Fatty Acids	DPAn-6	0.046519358	0.09588684
Fatty Acids	Adrenic acid	0.001847899	0.007942032
Fatty Acids	Citramalic acid	0.026013938	0.060400178
Fatty Acids	Oleic acid	0.829217154	0.900547661
Fatty Acids	2,2-Dimethylsuccinic acid	0.032313686	0.070659635
Fatty Acids	5Z-Dodecenoic acid	0.000145577	0.001225271
Fatty Acids	Ricinoleic acid	2.47979E-08	1.50295E-06
Fatty Acids	12-Hydroxystearic acid	0.21954796	0.349869682
Fatty Acids	Palmitelaidic acid	0.202784304	0.327699435
Fatty Acids	Linoelaidic acid	0.994733665	0.994733665
Fatty Acids	2,2-Dimethyladipic acid	0.522244089	0.643251865
Imidazoles	Imidazolepropionic acid	0.00022019	0.001533735
Indoles	Indolelactic acid	0.024109575	0.056629468
Indoles	Indole-3-pyruvic acid	0.13156914	0.231104054
Nucleotides	AMP	9.61462E-06	0.000121385
Nucleotides	GMP	0.047828875	0.09759023
Nucleotides	SAH	0.002116705	0.008726008
Organic Acids	Hydroxypropionic acid	0.001655326	0.007599453
Organic Acids	Lactic acid	0.001493617	0.007358796
Organic Acids	Malic acid	0.969444995	0.977480708
Organic Acids	Benzoic acid	0.393667505	0.537302946
Organic Acids	Fumaric acid	0.219967573	0.349869682
Organic Acids	Glutaric acid	0.000414011	0.002459715
Organic Acids	Aconitic acid	0.003224936	0.010505201
Organic Acids	alpha-Ketoisovaleric acid	0.196176725	0.322421072
Organic Acids	Ketoleucine	9.43975E-05	0.000866741

Organic Acids	3-Methyl-2-oxopentanoic acid	0.085052263	0.160565954
Organic Acids	Methylmalonic acid	0.001758148	0.007892131
Organic Acids	2-Hydroxyglutaric acid	0.593898872	0.714092692
Organic Acids	Guanidoacetic acid	0.09561394	0.178833481
Organic Acids	Shikimic acid	0.47146103	0.604773562
Organic Acids	3-Hydroxybutyric acid	0.473040707	0.604773562
Organic Acids	2-Hydroxybutyric acid	0.470299306	0.604773562
Organic Acids	Glutaconic acid	0.743971861	0.849052632
Organic Acids	Malonic acid	0.039759897	0.083661451
Organic Acids	Oxalic acid	0.007312803	0.020235427
Organic Acids	Maleic acid	0.73779183	0.846783805
Organic Acids	Acetoacetic acid	0.003095587	0.01025096
Organic Acids	Citric acid	0.002337953	0.009082048
Organic Acids	Isocitric acid	0.008124545	0.021594187
Organic Acids	Pyruvic acid	0.002337953	0.009082048
Organic Acids	Oxoglutaric acid	0.003562022	0.010901946
Organic Acids	Succinic acid	0.001422022	0.007181211
Organic Acids	alpha-Hydroxyisobutyric acid	0.804947275	0.890027799
Peptides	Anserine	0.810718391	0.890027799
Peptides	Carnosine	0.413523499	0.556878313
Peptides	Glycylproline	0.14264897	0.245784656
Peptides	Glycyleucine	0.048424112	0.097816707
Peptides	gamma-Glutamylalanine	0.004272223	0.012880435
Phenols	p-Hydroxymandelic acid	0.926502519	0.959761584
Phenols	Homovanillic acid	0.295647615	0.443356386
Phenylpropanoic Acids	Phenyllactic acid	0.002116705	0.008726008
Pyridines	Nicotinic acid	0.002681217	0.009523424
Pyridines	Picolinic acid	0.101643905	0.188367603
Pyridines	N-Methylnicotinamide	0.732794318	0.845854013
SCFAs	Ethylmethylacetic acid	0.003380386	0.010505201
SCFAs	Acetic acid	0.00773212	0.021106599
SCFAs	3-Hydroxyisovaleric acid	0.000379373	0.002322223
SCFAs	Propionic acid	0.247863497	0.385141742
SCFAs	Butyric acid	0.002753901	0.009523424
SCFAs	Isobutyric acid	0.014631489	0.036488405
SCFAs	Caproic acid	0.000183892	0.001397008
SCFAs	2-Methylpentanoic acid	0.509433515	0.631322516
